# Interleukin-33 and Obesity-Related Inflammation and Cancer

**DOI:** 10.3390/encyclopedia4040117

**Published:** 2024-11-23

**Authors:** Cameron Kowitt, Qiuyang Zhang

**Affiliations:** 1 Department of Structural & Cellular Biology, Tulane University School of Medicine, New Orleans, LA 70112, USA; 2 School of Public Health and Tropical Medicine, Tulane University, New Orleans, LA 70112, USA; 3 Tulane Center for Aging, Tulane University School of Medicine, New Orleans, LA 70112, USA; 4 Tulane Cancer Center, Tulane University School of Medicine, New Orleans, LA 70112, USA; 5 Louisiana Cancer Research Center, New Orleans, LA 70112, USA

**Keywords:** IL-33, ST2, immune response, inflammation, obesity, cancer

## Abstract

Interleukin-33 (IL-33) is a cytokine belonging to the IL-1 family. It is primarily associated with type 2 immune responses. It interacts with a receptor complex on immune cells in reaction to tissue damage or cellular injury. IL-33 is crucial in immune responses and is involved in various autoimmune and inflammatory diseases. Obesity is marked by chronic inflammation and is a known risk factor for several types of cancer. Recent studies have shown that IL-33 and its receptor complex are expressed in adipose (fat) tissue, suggesting they may play a role in obesity. While inflammation connects obesity and cancer, it is not yet clear whether IL-33 contributes to cancer associated with obesity. Depending on the cellular context, inflammatory environment, expression levels, and bioactivity, IL-33 can exhibit both protumorigenic and antitumorigenic effects. This review will explore the various functions of IL-33 in the inflammation linked to obesity and its relationship with cancer.

## 1. Introduction

Interleukins (ILs), a specific subgroup of cytokines [1,2] mainly produced and secreted by white blood cells (leukocytes) and other types of cells in the body, are essential for regulating inflammation, tissue repair, and immune responses in healthy tissue [3]. The human genome encodes over 50 interleukins and related proteins [4]. ILs also help to maintain the balance and function of normal cells and tissues. Importantly, they serve as vital chemical signals in the immune system, significantly influencing cancer development, progression, and treatment. Based on their structural homology, ILs are categorized into several protein families, including IL-1, IL-2, IL-6, IL-10, IL-12, and IL-17 [4]. The IL-1 family is further divided into three subfamilies (the IL-1 subfamily, the IL-18 subfamily, and the IL-36 subfamily) based on the length of the N-terminal pro-pieces. The IL-1 subfamily consists of IL-1α, IL-1β, the IL-1 receptor antagonist (IL-1Ra), and IL-33 [5].

IL-33, discovered in 2005 as a new member of the IL-1 family [6] and confirmed to be a Nuclear Factor from High Endothelial Venules (NF-HEVs) [7], has been linked to several health conditions. These include asthma, airway inflammatory diseases [8], organ fibrosis [9], kidney injury [10], pediatric heart disease and transplantation [11], rheumatoid arthritis [12,13], and neurodegenerative diseases [14]. Recent research involving knowledge mapping of IL-33 has revealed its essential role as a cytokine in numerous diseases, especially allergic diseases [15].

Overweight and obesity are significant health issues affecting millions of people worldwide [16]. According to the World Health Statistics 2024 [17], approximately 2.5 billion adults aged 18 and older are classified as overweight, including over 890 million individuals living with obesity. This statistic indicates that around 43% of adults (43% of men and 44% of women) are overweight, with 16% facing obesity. Epidemiological studies consistently identify obesity as a significant risk factor for 13 types of cancer [18], which together account for 40% of all cancer diagnoses in the United States each year. Globally, approximately 4–8% of all cancers are linked to obesity [19]. However, the causal relationship between obesity and cancer remains unclear. Research indicates that IL-33 plays a critical role in obesity [20-22] and shows promising findings related to IL-33 and various cancers [23-30]. Despite this, there is a lack of research on IL-33 in the context of obesity-related cancer, particularly regarding the mechanisms by which IL-33 influences these cancers.

Gaining insight into the complex connections between IL-33, obesity, and cancer could be crucial for the future management of these conditions, offering hope in the fight against these diseases. The mechanisms may involve IL-33’s regulation of specific immune cells related to obesity that contribute to disease aggressiveness. Understanding these potential pathways and targets could guide future research and therapeutic development. Therefore, the review will discuss the importance of researching obesity-related inflammation and cancer, and potential future directions.

## 2. The Biology of IL-33 and Its Receptors

IL-33, also known by several designations, including C9orf26 (Chromosome 9 Open Reading Frame 26), DVS27 (DVS27-Related Protein), NF-HEV (Nuclear Factor from High Endothelial Venules), DKFZp586H0523, NFEHEV, and RP11-575C20.2, is a cytokine that belongs to the IL-1 superfamily, identified explicitly as Interleukin-1 Family Member 11 (IL1F11) [6]. It was initially recognized for inducing type 2 immune responses [31,32], stimulating the production of immune cells involved in allergic reactions, and protecting against parasites. Understanding this function is crucial for comprehending its role in immune system regulation.

As a tissue-derived cytokine, IL-33 has various functions; it operates as a traditional cytokine and an intracellular nuclear factor [32,33]. Its association with tissue damage or necrosis is just one facet of its complexity. In response to harmful events such as infection, injury, or inflammation, barrier tissue cells, including epithelial cells, release IL-33. The released IL-33 plays a significant role in cell communication and regulating inflammation within the body.

### 2.1. IL-33

**Figure 1.**
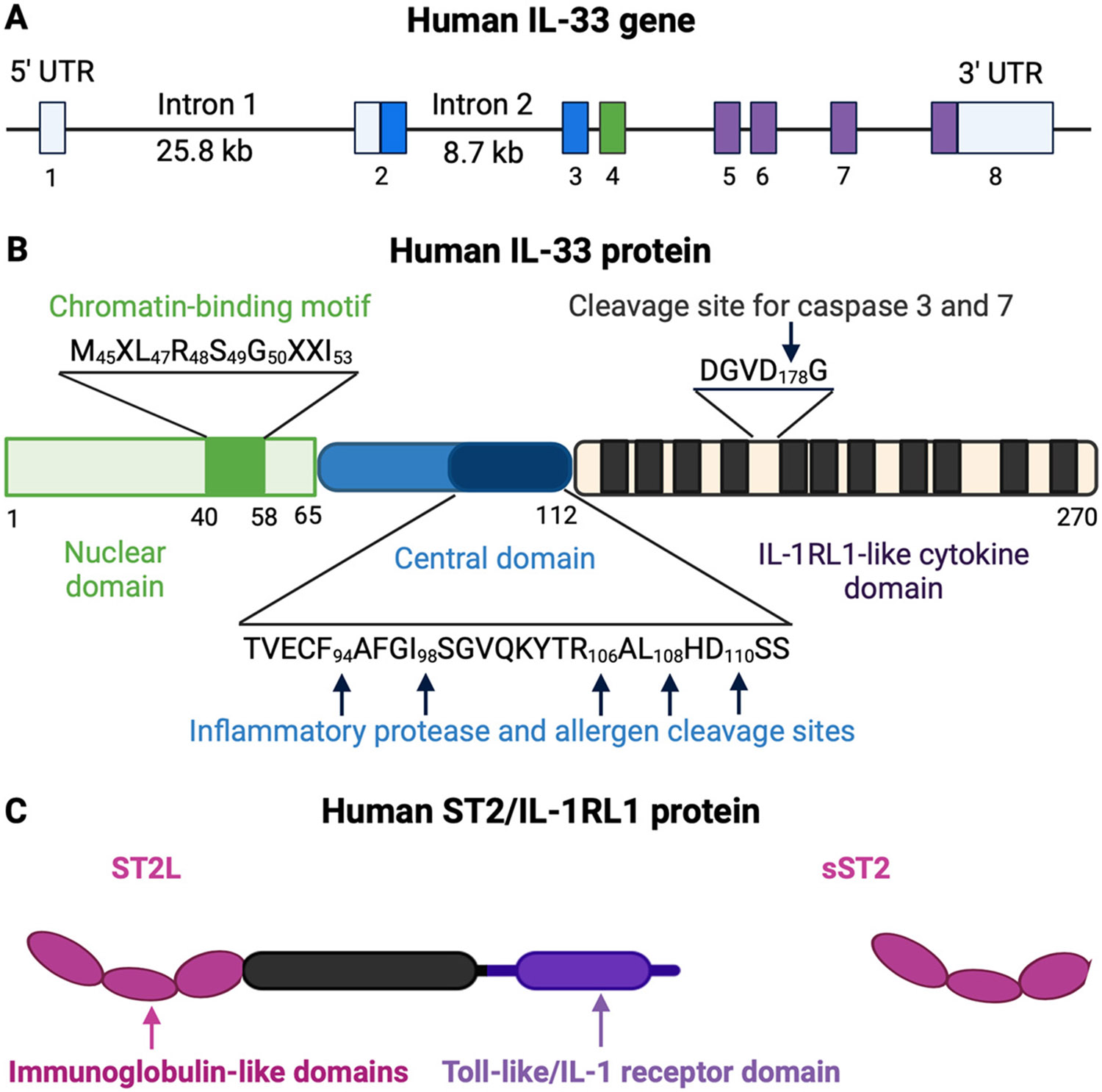
The human IL-33 gene and the IL-33 and ST2 proteins. (**A**) The human *IL33* gene is located on the short arm of chromosome 9 at 9p24.1. A large intron (25.8 kb; intron 1) separates the first non-coding exon (exon 1, also designated exon 1a) from the first coding exon (exon 2). An alternative exon 1b is located 4.6 kb upstream of exon 2. (**B**) Structure of the human IL-33 protein. It comprises two evolutionary conserved domains (the nuclear and IL-1-like cytokine domains) separated by a highly divergent linker region in the center (the central domain). Chromatin-binding motif and cleavage sites for caspases and inflammatory proteases are indicated. (**C**) The IL1RL1 gene encodes the ST2 protein. In humans, ST2 was identified in 3 splicing variants (not shown), but only the proteins ST2L and sST2 were identified in human cells [39].

The IL-33 gene is located on chromosome 9p24.1 in humans and chromosome 19qC1 in mice. The human IL-33 gene consists of eight exons, with exon 1 being non-coding and exons 2-8 coding for the IL-33 protein, spanning over 42kb of genomic DNA [34]. The IL-33 protein features an N-terminal domain containing a chromatin-binding motif, a nuclear localization signal, a central domain, and a C-terminal IL-1-like cytokine domain that gives IL-33 cytokine-like properties [6,32]. The nuclear localization signal can be found within amino acids 46-67 [35]. The ability to bind to DNA determines the functions of full-length IL-33 in the nucleus as a transcription factor [36]. In mice, two different promoters initiate IL-33 gene transcription, resulting in two IL-33 transcripts (Il33a and Il33b) with differing 5′-untranslated regions but encoding the same protein. The human IL-33 mRNA encodes a protein containing 270 amino acids, while the mouse IL-33 protein consists of 266 amino acids, with a 55% similarity between the two. Research indicates that the IL-33 protein is highly conserved among mammals and is closely related to IL-18 among the IL-1 family members. IL-33 has a three-dimensional structure akin to other family members, comprised of 12 β-strands arranged in a β-trefoil fold [37,38]. The binding area of IL-33 contains a mix of polar and nonpolar regions that facilitate specific interactions between the receptor and ligand [38] (Figure 1).

### 2.2. IL-33 Receptor

The IL-33 receptor, also known as serum stimulation-2 (ST2), interleukin 1 receptorlike 1 (IL1RL1), or T1, was initially discovered in 1989 and was considered an orphan receptor until the identification of IL-33 [40,41]. There are multiple isoforms of ST2, including the membrane-bound form (ST2L) and soluble form (sST2). ST2L consists of three immunoglobulin-like motifs responsible for extracellular ligand-binding (extracellular domain), an intracellular domain, and a transmembrane domain. The extracellular domain of ST2L binds IL-33 with the assistance of the IL-1 receptor accessory protein (IL-1RAcP), which forms the transmembrane IL-33 receptor complex and recruits downstream signaling components via its Toll/interleukin-1 receptor (TIR) domain [31]. Due to its lack of intracellular and transmembrane domains, but the possession of a C-terminal sequence [42], sST2 may serve as a decoy receptor for IL-33 [43]. The IL-1R8 receptor, known as SIGIRR (single immunoglobulin IL-1R-related receptor) or TIR8 [44], acts as a negative regulator of the IL-33/ST2 signaling pathway [45]. It can dimerize with ST2L to attenuate the biological effects of IL-33 (Figure 1).

### 2.3. IL-33/ST2 Signaling Pathway

IL-33 has a dual role, acting as a nuclear factor by binding to chromatin to suppress inflammatory responses and as a cytokine released into the extracellular space in response to cell damage or mechanical injury [33]. This dual functionality underscores its crucial role in immune modulation. IL-33 exerts its cytokine activity by binding to the primary receptor, ST2, and then recruiting the accessory receptor, IL-1RAcP [6,46-48].

When IL-33 binds to the IL-33 receptor, forming the ST2L and IL-1RAcP heterodimeric complex, it initiates a signaling pathway. Following receptor binding, the TIR domain dimerization starts a pathway that includes the myeloid differentiation primary response protein 88 (MyD88), IL-1R-associated kinase 1 (IRAK1), IRAK4, tumor necrosis factor (TNF) receptor-associated factor 6 (TRAF6), mitogen-activated protein kinases (MAPKs), or nuclear factor-κB (NF-κB) [34].

**Figure 2.**
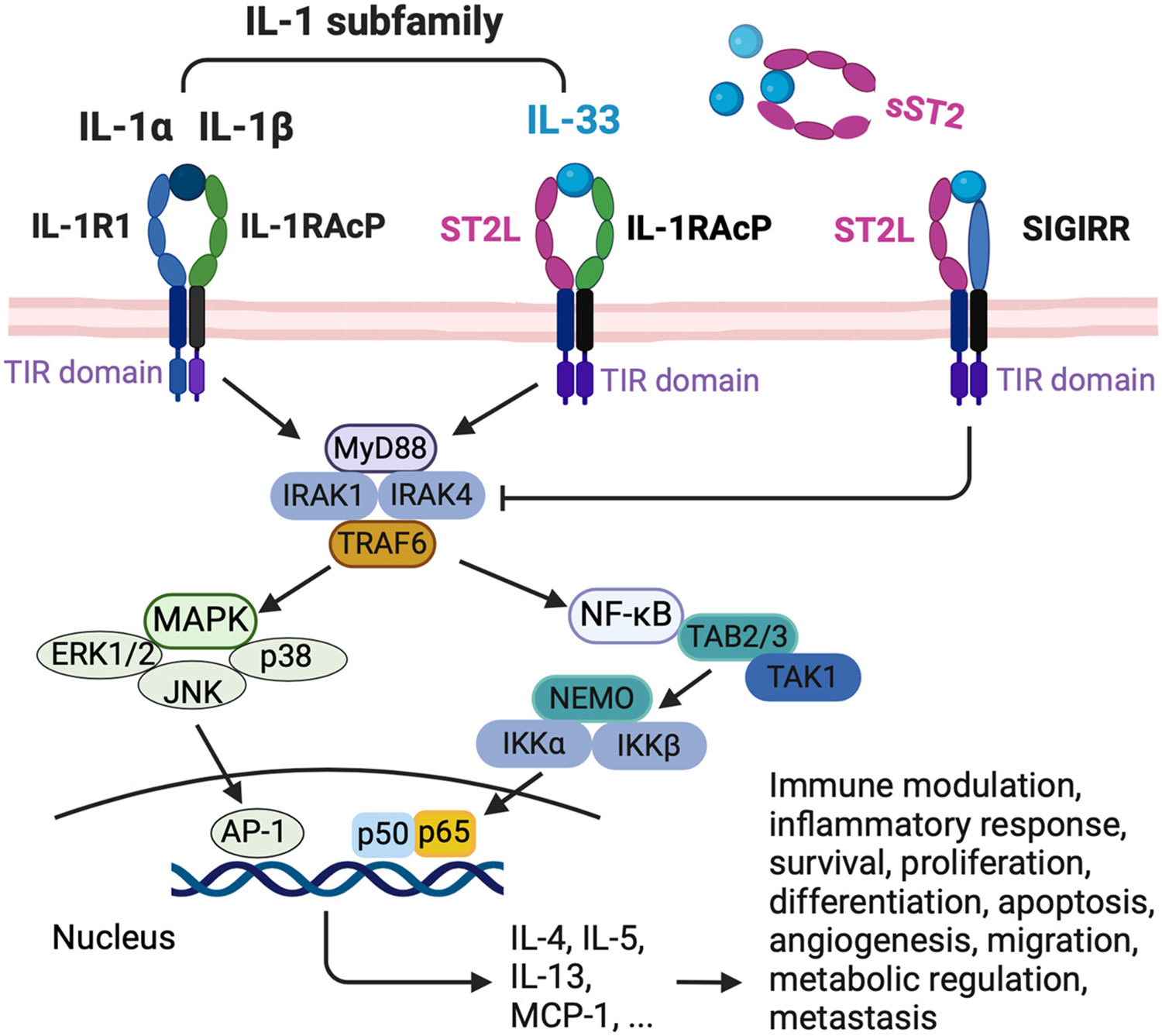
The IL-33/ST2 signaling pathway. When stromal cells experience damage or mechanical injury, they undergo necrosis and release IL-33. This cytokine activates the heterodimeric ST2/IL-1RAcP receptor complex on various immune cells. IL-33 can also bind to a decoy receptor composed of a soluble form of ST2 (sST2) or SIGIRR, which leads to inactivation. Upon activation of the ST2L, TIR initiates the pathway by first recruiting MyD88, which induces recruitment of IRAK1 and IRAK4, similar to the binding process seen with other interleukin-1 family members, such as Il-1α and IL-1β. This recruitment activates the transcription factor nuclear factor-κB (NF-κB) and the mitogen-activated protein kinase (MAPK) pathways. The activation is mediated by the MAPKs, including extracellular signal-regulated kinase (ERK), p38, and JUN N-terminal kinase (JNK), ultimately producing Th2 cytokines and chemokines. The activity of MAPK pathways and canonical NF-κB is regulated at multiple levels. ST2L, suppression of tumorigenicity 2 ligand; IL-1RAcP, IL-1 receptor accessory protein; MyD88, myeloid differentiation primary response protein 88; IRAK1, interleukin receptor-associated kinase 1; IRAK4, interleukin receptor-associated kinase 4;TRAF6, tumor necrosis factor receptor-associated factor 6; MAPK, mitogen-activated protein kinase; ERK1/2, extracellular signal-regulated kinases 1/2; JNK, c-Jun N-terminal kinase; p38, the subgroup of MAP kinases; AP-1, transcription factor; NF-κB, nuclear factor κB; TAK1, transforming growth factor (TGF)-β-activated protein kinase 1; NEMO, NF-κB essential modulator; IKKβ, inhibitor of κB (IκB) kinase β. This figure was created with BioRender.

The intracellular signaling pathways initiated by IL-33 activate a wide array of downstream effectors. These effectors play a significant role in immune modulation and inflammatory responses [49,50], cell survival [51,52], proliferation [27], differentiation [27], apoptosis [51], angiogenesis [53], migration [54], metabolic regulation [55], and metastasis [56] (Figure 2).

## 3. The Role of IL-33 in Immune Responses

### 3.1. IL-33 and ST2 Expression in Immune Cells

**Figure 3.**
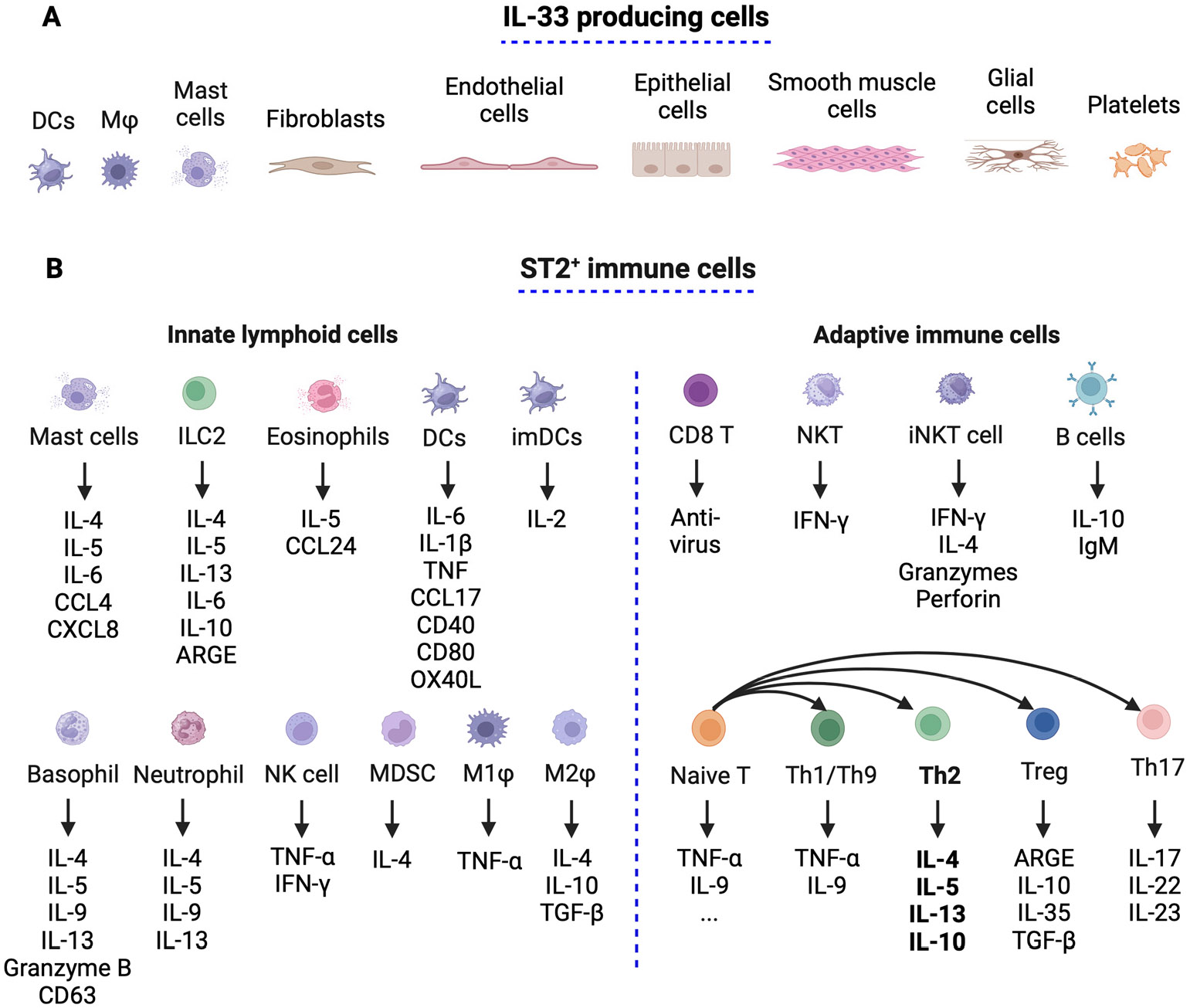
The production of IL-33 and the distribution of ST2. (**A**) Various immune cells release IL-33 in response to cell stress and injury. Under pathological conditions, IL-33 is released by endothelial and epithelial cells of barrier tissues such as the lung, intestine, skin, and fibroblasts, as well as glial cells, astrocytes, smooth muscle cells, platelets, and several types of immune cells, including macrophages (Mφ), dendritic cells (DCs), immature dendritic cells (imDCs), and mast cells. (**B**)Active IL-33 signals through ST2, expressed in different types of immune cells, including innate lymphoid cells and adaptive immune cells, generate different cytokines or polarize into the corresponding phenotypes in different pathological conditions. This figure was created with BioRender.

IL-33 is typically found in the stroma and is released by damaged or necrotic barrier cells such as endothelial and epithelial cells [57]. It is expressed at high levels in the steady-state nuclei of various cell types [34], including immune cells like resting dendritic cells (DCs) [58], activated macrophages [59], monocytes, and regulatory T cells (Tregs), as well as mast cells [59], endothelial cells, epithelial cells of barrier tissues such as the lung, intestine, skin, and fibroblasts, glial cells, astrocytes, smooth muscle cells, and platelets. IL-33 is a ubiquitous and crucial immune modulator that influences various immune cell responses [50]. It acts as an alarm signal (alarmins or danger signals), rapidly released from producing cells in response to cellular stress and injury [49] (Figure 3).

ST2 is expressed in T helper 2 (Th2) cells, mast cells [42,46,60], cardiomyocytes [61], and other immune cells [57,62], including innate lymphoid cell type 2 (ILC2) [63], Tregs [64], CD4^+^ and CD8^+^ T cells [65], NKT cells [66,67], and myeloid cells, including basophils, eosinophils, and macrophages [59]. Recently, ST2 was reported to be expressed in Th1 cells [68,69], though the expression level was low and transient upon virus infection [70] (Figure 3).

### 3.2. Il-33’s Role in Immune Responses

The IL-33/ST2 pathway plays a crucial role in Th2 responses. When IL-33 activates Th2 receptors, it produces IL-4, IL-5, and IL-13. These cytokines, produced by Th2 cells, enhance chemokine production in epithelial cells [71-73]. The IL-33 gene and these cytokines are linked to Th2-related diseases, protecting against intracellular pathogens, promoting tissue repair, and contributing to chronic inflammatory diseases [74].

IL-4, a critical Th2 cytokine, is primarily produced by activated T cells, mast cells, basophils, and eosinophils. Its primary function is to transform naive CD4^+^T cells into Th2 cells, which initiates allergic reactions and triggers immune responses against extracellular parasites [75]. IL-4 also affects various diseases, particularly inflammatory bowel disease (IBD), where it contributes to chronic inflammation and fibrosis by promoting tissue remodeling and scarring, as well as autoimmune diseases [73]. Moreover, IL-4 supports the proliferation and survival of several cancer cells [76], being overexpressed in many human tumor types, including malignant glioma, ovarian, lung, breast, pancreatic, colon, and bladder carcinomas, which also overexpress its receptors (IL-4R) [77-79]. While several therapeutics have been developed for asthma and inflammatory diseases, their lack of efficacy has hindered further development. However, most of these treatments have shown tolerance and high safety in humans, making clinical trials for cancer therapy a possibility [76].

IL-5 is significant in developing allergic reactions and inflammation, as it regulates the production, maturation, activation, and survival of eosinophil progenitors in the blood [80]. It stimulates B lymphocytes to produce more antibodies and increases the number of eosinophils in the airways. Its primary role is to promote the activation and longevity of eosinophils, aiding their migration from the blood to the airways [81]. By encouraging the production of antibodies, particularly immunoglobulin A (IgA), which is crucial for mucosal immunity, IL-5 also influences B cell activity [81]. Additionally, IL-5 acts as a biological modifier that can enhance the immune system in cancer therapy.

IL-13 is a critical factor in developing allergic inflammation [82]. It closely interacts with IL-5 and significantly impacts the airways by promoting mucus production [83]. IL-13 encourages goblet cells in the airway to produce mucus, leading to airway blockage and mucus plugging, which directly contribute to asthma [83,84]. Furthermore, IL-13 is overexpressed in various solid tumors and is associated with poor prognosis in conditions such as glioblastoma, colorectal cancer, adrenocortical carcinoma, pancreatic cancer, and breast cancer [85].

## 4. IL-33 in Obesity

Obesity is linked to chronic inflammation, hormonal imbalances, insulin resistance, high insulin levels, adipokines changes, and immune function alterations [86]. Hormones such as adipokines, produced by adipose tissue, contribute to low-grade inflammation, leading to conditions like insulin resistance, diabetes, high blood pressure, and asthma, all commonly associated with obesity [87]. These adipokines can stimulate cell growth, angiogenesis, and apoptosis, all directly connected to cancer progression. Furthermore, these hormonal functions regulate the appetite, energy metabolism, and inflammation, increasing individuals’ susceptibility to cancer development and progression [88].

Adipose tissue is essential for regulating energy balance and is generally categorized into white adipose tissue (WAT) and brown adipose tissue (BAT) [89-91]. Additionally, there is a type of adipose tissue known as brown-in-white or (brite)/beige adipose tissue (BeAT), which shares the characteristics of both WAT and BAT in terms of morphology and function [92]. Research on the role of IL-33 in developing metabolic disorders such as diabetes, obesity, and cardiovascular disease has produced mixed results [22]. However, the IL-33/ST2 pathway is thought to be protective during obesity. Manipulating IL-33 expression to induce Th2 cytokines and promote macrophage polarization may offer a promising therapeutic strategy for treating or preventing type 2 diabetes in obese patients.

### 4.1. IL-33 and ST2 Immune Cell Distribution in Adipose Tissue

Immune cells and their mediators significantly regulate the metabolism, leading to a new understanding of immune regulation in metabolic processes [21]. Innate immune cells present in adipose tissue include macrophages, neutrophils, eosinophils, dendritic cells (DCs), innate lymphoid cells (ILCs), and natural killer (NK) cells [93]. In lean adipose tissue, the most common immune cells are alternatively activated macrophages (M2-like macrophages), type 2 innate lymphoid cells (ILC2s), eosinophils, Tregs, and Th2 cells [94,95]. These cells contribute to an anti-inflammatory environment.

However, during obesity development and adipocyte hypertrophy, the distribution of immune cell populations changes. Most immune cells increase in adipose tissue, leading to an inflammatory state that supports insulin resistance. Exceptions to this trend include Tregs, eosinophils [21], and Th2 cells [96].

**Figure 4.**
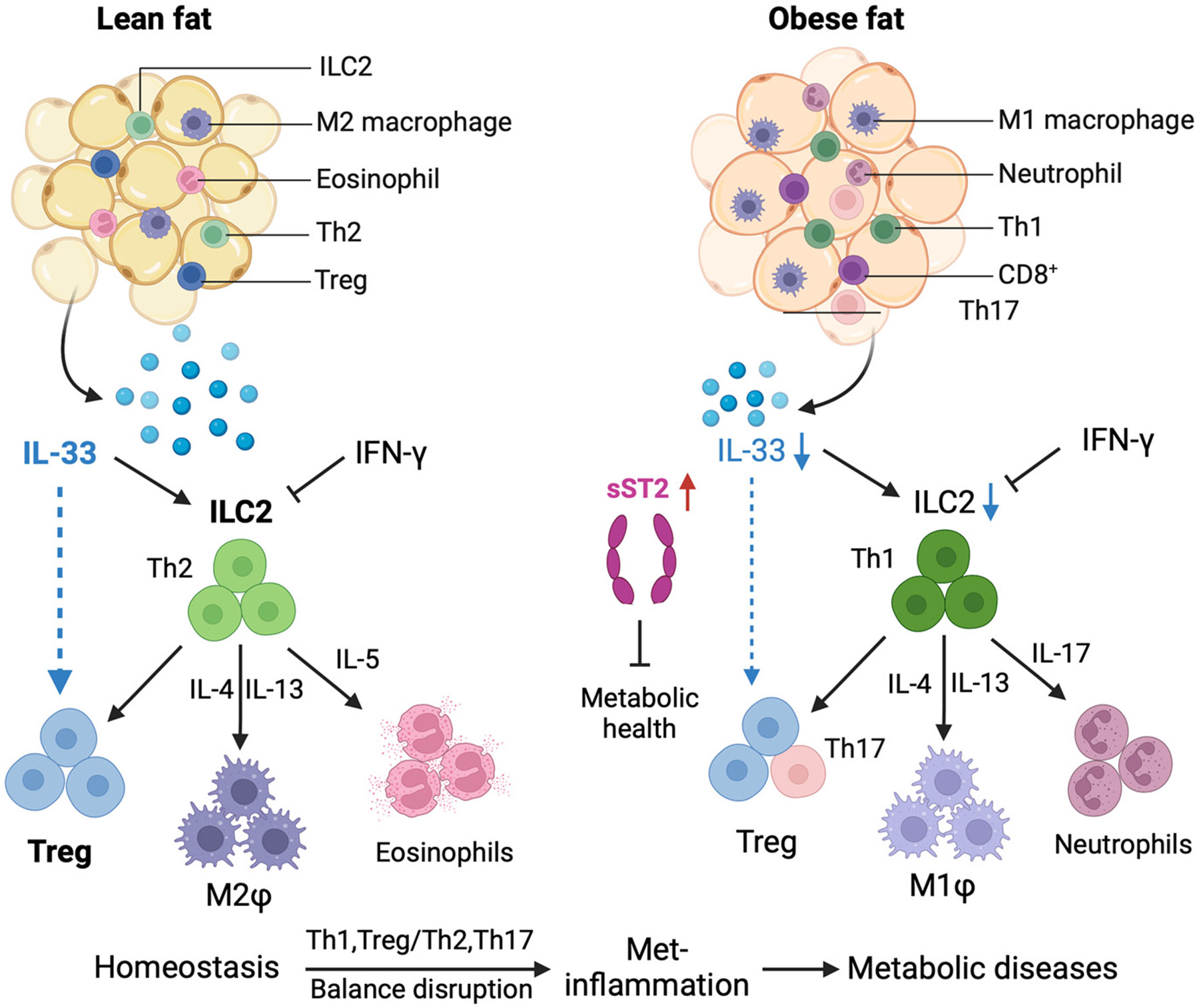
IL-33 and ST2 immune cells in adipose tissue. During the development of obesity, adipocyte hypertrophy is accompanied by significant changes in immune cell populations. Most immune cells increase in quantity in adipose tissue due to obesity, with a few exceptions, including regulatory T cells (Tregs) and eosinophils. Tregs and eosinophils reduce the inflammatory responses of other immune cells in adipose tissue, particularly adipose tissue macrophages. As obesity progresses, the population of IL-33/ST2 target immune cells in adipose tissue also varies, leading to obesity-related inflammation. Lean adipose tissue primarily contains non-inflammatory cells, such as activated M2 macrophages (M2φ), eosinophils, ILC2 cells, Tregs, and Th2 cells. In contrast, obesity shifts the immune profile of adipose tissue toward a pro-inflammatory state, characterized by an influx of macrophages (M1φ), NK cells, neutrophils, CD4^+^ T cells (Th1, Th17), and CD8^+^ T cells. When IL-33 is reduced during obesity, sST2 is increased [108], which can inhibit metabolic health. In addition, the balance between Th1 and Th2 cells, and between Treg and Th17 cells, is disrupted, leading to increased adipose tissue-related inflammation (often referred to as “met-inflammation”) and contributing to the development of metabolic diseases. DC, dendritic cell; M, macrophage; ILC2, innate lymphoid cell type 2; iNKT, invariant natural killer T; NK, natural killer cells; NKT, natural killer T cells; MDSC, myeloid-derived suppressor cells. This figure was created with BioRender.

IL-33 and its receptor ST2 are abundant in adipose tissue, including preadipocytes, adipocytes, and endothelial cells [97-99]. Additionally, infiltrating immune cells that express IL-33 and ST2 are plentiful in this tissue (Figure 4).

### 4.2. Effect of IL-33 in Adipose Tissue During Obesity

Despite its increased expression in obesity, IL-33 alone cannot maintain homeostasis in obese adipose tissue. Research indicates that IL-33 treatment can improve the inflammation and metabolic changes related to obesity [22]. The role of IL-33 in immunity and the metabolism is complex, demonstrating both pro- and anti-inflammatory properties [22]. IL-33 is essential for maintaining adipose tissue homeostasis and may help protect against obesity and type 2 diabetes [100,101].

Tissue-resident stromal cells produce IL-33, supporting immune homeostasis in adipose tissue through the involvement of ILCs [101]. Furthermore, IL-33 protects against adipose tissue inflammation during obesity by inducing Th2 cytokines in WAT and promoting the polarization of WAT macrophages toward an M2, an alternatively activated phenotype [100,102,103]. This process reduces adipose mass and lowers fasting glucose levels [100]. In laboratory cultures of WAT, IL-33 stimulates the production of Th2 cytokines and chemokines while increasing the serum levels of Th2 cytokines and the number of Th2 cells in WAT. This shift affects macrophage activity in both adipose and liver tissues, reducing the chronic inflammatory response associated with obesity. Additionally, adiponectin can inhibit IL-33 signaling through AMPK-mediated feedback [104].

As obesity progresses, adipocyte hypertrophy significantly alters immune cell populations. While WAT shows increased IL-33 expression, local ILC2 levels tend to decrease [22]. This imbalance highlights the need to understand the discrepancies in regulating IL-33 at both tissue-specific and systemic levels amid obesity [22]. IFN-γ inhibits the activation and proliferation of ILC2 cells, limiting the functions of IL-33-mediated ILC2 and Tregs while promoting a Th1 immune response [97]. This suppression by IFN-γ likely facilitates inflammatory responses and reallocates metabolic resources necessary for host protection (Figure 4).

Moreover, obesity is linked to an imbalance between Th17 and Treg cells, which can lead to metabolic disorders [104-106]. It also disrupts the Th1/Th2 balance, increasing the number of Th1 cells in adipose tissue while decreasing Th2 cells [107]. Although there are currently no direct studies examining how IL-33 contributes to this imbalance, these disruptions may result from reduced levels of IL-33, warranting further investigation (Figure 4).

## 5. Effect of Il-33 in the Tumor Microenvironment

The cytokine IL-33 has a dual role in the tumor microenvironment (TME), promoting and inhibiting tumor growth. Its protumor effects involve the accumulation of immune-suppressive cells. Conversely, IL-33 can inhibit tumor growth by interacting with the T cell receptor (TCR) and IL-12 signaling, enhancing the effectiveness of CD8^+^ T cells [109]. Additionally, IL-33 amplifies the responses of Th1 CD4^+^ T cells and Th2-type cells by acting on various immune cells, including human basophils, allergen-reactive Th2 cells, iNKT, NK cells [110], eosinophils, and ILC2 [111].

### 5.1. IL-33 and ST2 Immune Cell Distribution in the Tumor Microenvironment (TME)

The TME varies among different types of tumors but generally includes immune cells, stromal cells, blood vessels, and the extracellular matrix [112], which actively promote cancer progression. Depending on the context, immune cells can play a pivotal role in inhibiting or supporting tumor growth [113]. ST2 is expressed in various immune cells,and IL-33 is released from damaged structural cells. IL-33/ST2 signaling is involved in numerous cancers, exhibiting both protumor and antitumor functions [114].

Understanding the diverse effects of IL-33 on immune responses in the cancer context is crucial [115]. For example, IL-33 can activate ILC2 in the lung, which promotes tumor burden by leading to innate type 2 inflammation and suppressing the production of IFN-γ and the cytotoxic functions of lung NK cells [116]. In melanoma, the overexpression of IL-33 has been shown to inhibit lung metastases by activating CD8^+^ T cells and NK cells [117]. Additionally, the induced expression of IL-33 in tumors can enhance anti-melanoma immune responses via IFN-γ-producing CD8^+^ T cells and NK cells [118], presenting potential opportunities for cancer therapy.

Eosinophils [119] and DCs [120] may also mediate the anti-melanoma effects of exogenous IL-33. In breast cancer, IL-33 secreted by cancer-associated fibroblasts enhances ILC2 and Th2 type responses, induces the TCR-independent secretion of IL-13, and recruits immunosuppressive granulocytes [121]. Furthermore, IL-33 activates the intrinsic signaling pathway in Treg cells, which is necessary for their immunosuppressive action in cancer [122]. When combined with the pro-inflammatory cytokine IL-12, IL-33 amplifies the production of the Th1 cytokine IFN-γ [109].

**Figure 5.**
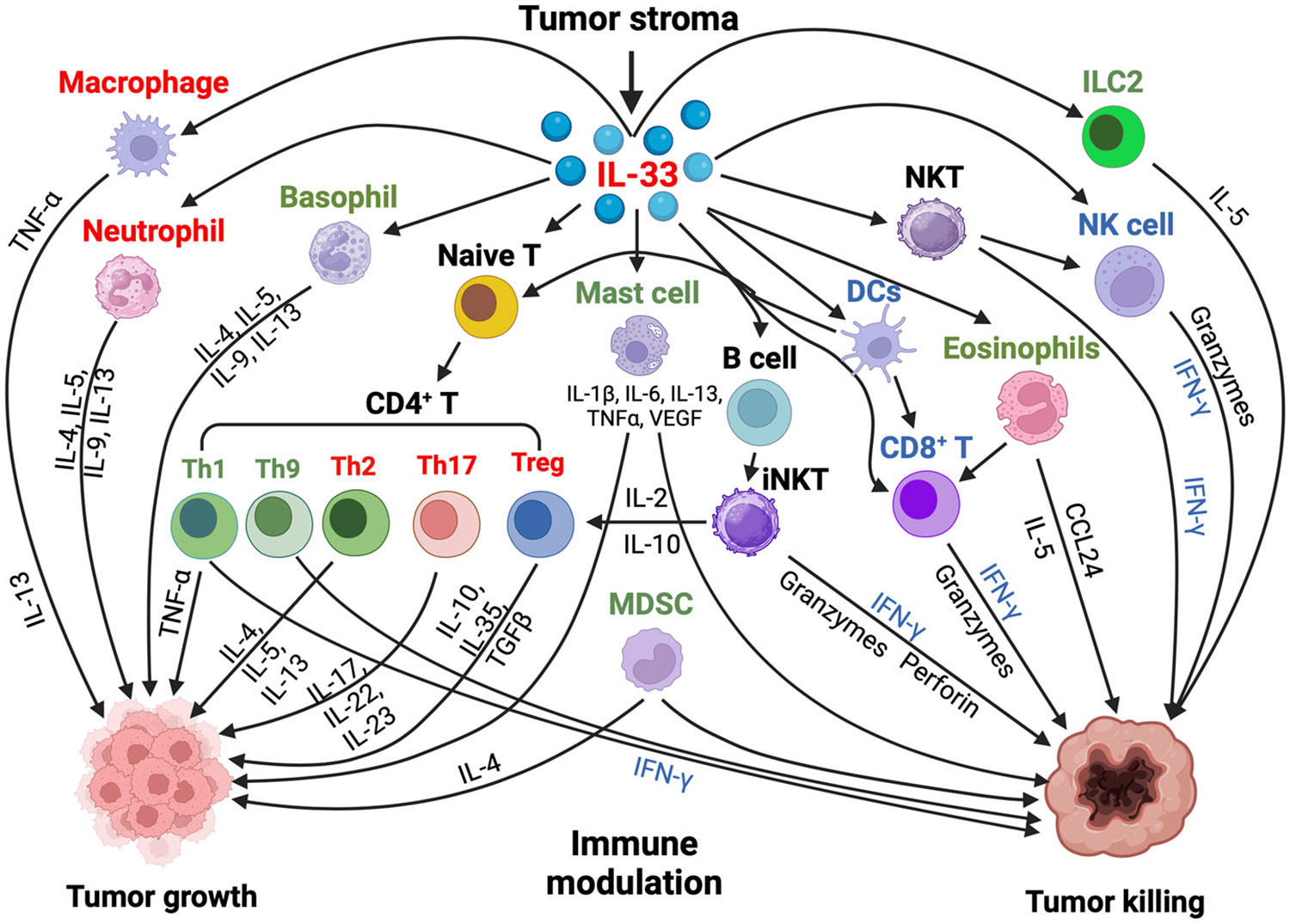
Role of IL-33 in the tumor microenvironment: IL-33 influences immune regulation and can have both protumor and antitumor effects. The population of immune cells targeted by IL-33/ST2 in the TME changes depending on the context. This results in opposing effects in different tumors. Along with tumor development, IL-33 is likely downregulated in epithelial cells but upregulated in the tumor microenvironment. The increased IL-33 expression in stroma either maintains or activates suppressor immune cells such as macrophages, Tregs, and CD4^+^ Th2 or Th17 cells, thus contributing to tumor growth and metastasis. However, IL-33 may also have an antitumor effect by activating innate natural killer (NK) cells and adaptive (CD4^+^ Th1 or CD8^+^ T cells) immune responses. IL-33/ST2 signaling can lead to a dual role on other cell types such as eosinophils, basophils, group 2 innate lymphoid cells (ILC2s), and myeloid-derived suppressor cells (MDSCs) either directly or through interaction with other cell types, depending on cancer type [123]. Cell names in red indicate that their primary function is a protumor effect; cell names in blue indicate that their primary function is an antitumor effect; cell names in green indicate dual roles. The cells are named in black, and their primary functions are on the corresponding side. This figure was created with BioRender.

Overall, the IL-33/ST2 signaling pathway significantly influences the TME in cancer by impacting immune effector cells and regulating the recruitment of cells that either promote or inhibit tumor growth [123]. Due to its immunomodulatory properties, IL-33 has the potential to synergize with various cancer therapies, including immune checkpoint blockade and chemotherapy. This information focuses on the effects of IL-33/ST2 on immune cells and stroma cells within the TME [111] (Figure 5).

### 5.2. Effect of Il-33 on Neutrophils, Eosinophils, Mast Cells, and Basophils

Depending on the specific disease context, IL-33 affects innate immune cells, including neutrophils, eosinophils, mast cells, and basophils [124]. It can promote inflammatory or regulatory responses in neutrophils and eosinophils, contributing to tissue repair or immunosuppression [125]. Studies have demonstrated that IL-33 directly influences murine neutrophils, enhancing their recruitment and activation in inflamed tissues [125]. In cancer, IL-33-driven neutrophils are associated with pro-inflammatory and regulatory functions.

Eosinophils, which play a vital role in the host defense against parasites and infections, are activated by IL-33 for allergic reactions and tissue repair [125]. In the context of cancer, Il-33 is linked to using eosinophils for antitumor effects, such as inhibiting growth and preventing metastasis. IL-33-activated eosinophils express higher effector molecules, including the eosinophil cationic protein (ECP), eosinophil peroxidase (EPX), and granzymes. They also express integrin CD11b/CD18, which forms immune synapses that facilitate the killing of tumor cells in vitro and in vivo [126].

Mast cells, which respond to IL-33, accumulate in tumors and their microenvironment during disease progression [127]. They are involved in tumor growth, with their mediators exhibiting both pro- and antitumorigenic roles in various human cancers [128]. Activating mast cells and basophils by IL-33 may influence tumor progression by modulating cytokine release and triggering immune complex activation. Recent studies indicate that tumorinfiltrating mast cells activated by IL-33 secrete IL-2 and promote the expansion of ICOS^+^ Tregs, which contributes to gastric cancer progression [129].

Basophils, produced in the bone marrow, are white blood cells needed for maintaining a healthy immune system. Abnormal levels of basophils can indicate conditions such as inflammation and hyperthyroidism. IL-33 can activate both murine and human basophils, increasing the production of histamine and cytokine in vitro and promoting their expansion in vivo [130-133]. However, a limited understanding of the relationship between basophils, cancer, and IL-33 regulation remains [125,134].

### 5.3. Effect of Il-33 on Macrophages and Dendritic Cells

Macrophages and DCs play crucial roles in the innate immune system, particularly regarding tumor immunity and immunology [135]. IL-33 is a crucial regulator of the activity of DCs and macrophages within the TME, displaying both pro- and antitumor effects [135]. Notably, IL-33 is essential for promoting M2-like polarization in macrophages, a process associated with immunosuppressive and tumor-promoting properties [135]. This polarization involves the release of anti-inflammatory cytokines and the stimulation of tumor angiogenesis, invasion, and metastasis [134,136].

In addition to its effects on macrophages, IL-33 also influences DCs. DCs activated by IL-33 demonstrate increased cytokine production and enhanced antigen presentation capabilities, which promote T cell activation and bolster antitumor immune responses. However, conflicting studies regarding IL-33’s influence on DC-mediated antitumor immunity suggest that its function in DCs may vary based on its location within the body [134]. This discrepancy highlights the need for further research in this area.

Moreover, with the assistance of dectin1-activated DCs, IL-33 can induce Th9 cell differentiation and enhance the antitumor efficacy [137,138]. While some studies indicate that IL-33 improves T cell activation and DC-mediated antigen presentation, others argue that IL-33 may lead DCs to acquire tolerogenic characteristics, inhibiting the immune system’s ability to combat cancer [134].

### 5.4. Effect of Il-33 on CD8^+^ T, NK, and NKT Cells

Several studies have demonstrated that IL-33 expression is positively correlated with the recruitment of CD8^+^ T cells and NK cells within the TME. CD8^+^ T, NK, and NKT cells are effective tumor-killer cells with many phenotypes and functions relevant to antitumor immunity [139]. IL-33 directly activates NK and NKT cells, leading to the production of IFN-γ and the promotion of Th1 immunity [140]. It also targets immunoregulatory invariant NKT (iNKT) cells to enhance their activation state [66]. This activation induces the production of IFN-γ and promotes Th1 immunity in both human and mouse NK cells [138].

When combined with IL-12, IL-33 further enhances NK cell activation, improving their ability to destroy tumor cells through cytotoxicity. In preclinical models, IL-33 has been shown to support the recruitment and activation of cytotoxic NK cells in the TME, which hinders the formation of metastasis and suppresses tumor progression [134]. However, it is crucial to note that conflicting reports are suggesting that, in specific tumor microenvironments, IL-33/ST2 signaling may hinder NK cell activation. This underscores the need to consider the context-dependent effects of IL-33 on NK cell-mediated antitumor responses. Such considerations should be a key focus in future research and clinical applications, emphasizing the necessity for further investigation and careful application [27].

### 5.5. Effect of Il-33 on Myeloid-Derived Suppressor Cells

IL-33 significantly affects myeloid-derived suppressor cells (MDSCs), which are critical mediators of immune suppression within the tumor [23]. MDSCs play a crucial role in cancer by helping tumors evade the immune system and accelerating disease progression [23]. IL-33 partially regulates the function and development of MDSCs within the TME [23]. In this environment, IL-33 reduces apoptosis and supports MDSC survival by inducing autocrine secretion of GM-CSF. This creates a positive feedback loop that promotes MDSC accumulation [23]. The increase and promotion of MDSCs are linked to tumor development and heightened immunosuppression due to IL-33. However, studies have shown that, in some contexts, IL-33 can inhibit the growth of MDSCs and decrease their immunosuppressive capabilities, resulting in enhanced antitumor immune responses and tumor regression [23,134].

### 5.6. Effect of Il-33 on T Helper Cells

IL-33 plays a significant role in regulating the development and function of CD4^+^ T cells, including CD4^+^ T helper (Th) cells and CD4^+^ Treg cells [124,141]. Early in T cell activation, IL-33 stimulates Th1 differentiation and works alongside IL-12 to enhance Th1 polarization [124,141]. Moreover, IL-33 may promote Th1 cell differentiation depending on the presence of IL-12 and ST2 [141]. MyD88 is essential for IL-33, as well as IL-12-induced Th1 cell development and the production of IFN-γ [141]. Additionally, IL-33 fosters the proliferation of Th cells that produce IL-9 (Th9), strengthening immune responses against tumors.

Conversely, IL-33 signaling encourages the proliferation of suppressive CD4^+^ Foxp3^+^ GATA3^+^ Treg cells, especially in tumor-specific environments high in IL-33 [60,141]. These Treg cells have a complex regulatory role, which may limit antitumor immunity while exhibiting suppressive solid activities [134]. In preclinical models, IL-9-producing Th9 cells are vital for initiating robust immune responses against tumors and promoting tumor regression. Research on T cell differentiation indicates that IL-33 has context-dependent effects on Th1- and Th2-skewing, reflecting the complexities of immune system regulation [134,141]. The unique cues from the tumor microenvironment can influence how these signaling pathway function in Th cell development.

Th17 cells play a significant role in inflammation and are implicated in various autoimmune diseases [142]. Their activity is influenced by different molecular signals within the TME [143], which affects their proliferation, differentiation, metabolic reprogramming, and phenotypic transformation, resulting in a dual impact on tumor progression [143]. Recent studies suggest that Th17 cells accumulating in the small intestine during inflammation express the IL-33 receptor (ST2), with intestinal epithelial cells (IECs) being the primary source of IL-33 [144]. When exposed to IL-33, both mouse and human Th17 cells exhibit reduced expression of pro-inflammatory genes and increased IL-10. This anti-inflammatory cytokine can limit intestinal inflammation and control previously activated pathogenic Th17 cells. The findings indicate that, in response to IL-33, Th17 cells acquire immunosuppressive properties. The IL-33/ST2 axis is likely crucial in regulating pathogenic Th17 cells in the small intestine, helping to maintain homeostasis [144].

### 5.7. Effect of Il-33 on CD4^+^ Treg Cells and in Tumor Immunity

IL-33 signaling influences effector Th cells and the growth and activity of Treg cells within the TME. Research shows that suppressive CD4^+^ Foxp3^+^ GATA3^+^ Treg cells proliferate in response to ST2/IL-33 signaling, both in vivo and in vitro, which aids in immunosuppression and tumor immune evasion [134]. IL-33 signaling promotes the generation of IL-2 by DCs and facilitates TGF-β1-mediated Treg cell development, leading to the expansion of ST2^+^ Treg cells [134,145]. These IL-33-expanded Treg cells are present in both immune and non-immune tissues and exert a strong suppressor effect, creating an immunosuppressive tumor microenvironment [146].

In the lungs, CD4^+^Foxp3^+^ Treg cells are expressed the IL-33 receptor ST2 [147]. When exposed to IL-33, Treg cells increase their canonical Th2 transcription factor GATA3 and ST2 expression levels, along with producing type 2 cytokines. This exposure leads to a significant shift in the immune response, causing Treg cells to lose their ability to suppress effector T cells [147]. Furthermore, IL-33 has been implicated in promoting the accumulation and maintenance of ST2^+^ Treg cells in inflamed tissues, exacerbating immunosuppression and facilitating tumor progression [134].

The complex interaction between IL-33 signaling and CD4^+^ T cell subsets underscores its significance in supporting antitumor immune responses [134]. By stimulating the growth of suppressive Treg cells and influencing Th cell differentiation, IL-33 regulates adaptive immune responses within the tumor. The crucial role of IL-33 in promoting Treg cell expansion highlights its potential as a target for therapeutic intervention in cancer immunotherapy, emphasizing the importance of understanding and utilizing IL-33 in cancer treatment [138].

### 5.8. IL-33-Expressing Cells and the Effects of IL-33 on Non-Immune Cells in the TME

The impact of IL-33 is influenced by the types of cells that produce it within the TME. These cells include immune cells, stromal cells, non-tumor epithelial cells, and transformed (epithelial) tumor cells, which are significant sources of IL-33 in the TME [57]. Fibroblasts, in particular, play a crucial role; their dysregulation during cancer creates an environment that fosters tumor growth by remodeling the extracellular matrix and releasing pro-angiogenic factors [148,149]. Research has shown that cancer-associated fibroblasts can induce IL-33 production [150].

Stromal cells have also been identified as a primary source of IL-33 among non-immune cells, and IL-33 derived from these cells has been linked to tumor metastasis [115,148,151,152]. The analysis of The Cancer Genome Atlas (TCGA) data and mRNA sequencing from the TCGA revealed that IL-33, expressed in endothelial and epithelial cells, could further contribute to tumor immune responses [115,151]. Moreover, IL-33 produced by non-tumor epithelial cells has been shown to drive colorectal cancer tumorigenesis in APC^Min/+^ mice, which serve as an excellent model for colorectal tumors due to a mutation in the APC gene, a significant tumor-suppressor gene in the Wnt signaling pathway [153].

The expression of IL-33 changes during tumor progression. At the same time, it is highly expressed in normal epithelial cells, and tumor development leads to its downregulation in epithelial cells and upregulation in the tumor stroma and serum [114]. Therapeutic overexpression of IL-33 in tumor epithelial cells promotes type 1 antitumor immune responses through CD8^+^ T cells and NK cells. In contrast, IL-33 from tumor stroma fosters immune tolerance and suppression via Tregs and MDSCs [114]. Overall, IL-33 expression is context-dependent. In some tumors, IL-33 is highly expressed in normal epithelial cells but downregulated in cancer cells in advanced conditions [114,154,155]. Conversely, IL-33 or ST2 protein expression levels in other tumors increase in cancerous lesions compared to matched normal tissues [123,156,157].

## 6. Discussion of IL-33 and Obesity-Associated Cancer

Recent scientific research has confirmed a clear connection between a high BMI and the risk of developing various types of cancer [19,30]. While obesity itself may not directly cause cancer, it increases the likelihood of developing aggressive, fast-growing forms of cancer that are more challenging to treat. Therefore, addressing obesity is vital for reducing the risk of cancer-related complications [19].

Research suggests that inflammation is connected to obesity and cancer [158]. IL-33 plays multiple roles in adipose tissue and the TME. However, studies explicitly examining the direct effects of IL-33 in obesity-related cancer are limited. Given its significant role in both obesity and cancer, more research is needed to fully understand IL-33’s potential in regulating obesity and decreasing cancer risk. Current findings are promising and may indicate a new pathway for disease management.

Future research should investigate how IL-33 impacts fat tissue function, energy expenditure, and immune modulation to identify potential therapeutic targets. It is also crucial to further explore the influence of immune cells on both antitumor and protumor processes in different types of obesity-related cancers. Targeting IL-33 signaling pathways and their downstream effects could help regulate obesity while exhibiting antitumor properties. Additionally, IL-33’s anti-inflammatory characteristics provide another possible intervention avenue, as chronic inflammation is a critical factor in obesity and a significant contributor to cancer development. Therapies based on IL-33 could reduce obesity-induced inflammation in fat tissue and other affected organs, subsequently lowering cancer risk.

Moreover, conducting clinical trials and utilizing personalized medicine approaches are essential to evaluating the safety and effectiveness of IL-33-based treatments. This process involves developing tailored treatment plans considering each patient’s unique risk factors and metabolic profiles. A comprehensive understanding of IL-33’s role in immune regulation and metabolism is critical for harnessing it as a therapeutic target to manage obesity and mitigate cancer risk. Collaboration among scientists, clinicians, and industry partners is vital for expanding our understanding of IL-33 biology and translating scientific discoveries into practical applications.

## 7. Conclusions and Prospects

Research into the various roles of IL-33 in immune modulation, obesity, and cancer is essential. The complex interactions between the IL-33/ST2 axis in adipose tissue and the tumor microenvironment (TME) deserve a thorough investigation, especially concerning obesity-related tumors. Given IL-33’s importance in both obesity and cancer, much remains to be explored regarding the roles of the IL-33/ST2 pathway in various types of obesity-related cancers and their responses to treatment. Both animal and human studies are necessary to fully understand how the IL-33/ST2 axis contributes to these cancers.

## Data Availability

The data presented are available in the references cited in this article.

## References

[1] Takeuchi T. Cytokines and cytokine receptors as targets of immune-mediated inflammatory diseases-RA as a role model. Inflamm. Regen 2022, 42, 35. 36451227 10.1186/s41232-022-00221-x PMC9713106

[2] Turner MD; Nedjai B; Hurst T; Pennington DJ Cytokines and chemokines: At the crossroads of cell signalling and inflammatory disease. Biochim. Biophys. Acta 2014, 1843, 2563–2582. 24892271 10.1016/j.bbamcr.2014.05.014

[3] Samadi M; Kamrani A; Nasiri H; Shomali N; Heris JA; Shahabi P; Ghahremanzadeh K; Mohammadinasab R; Sadeghi M; Sadeghvand S; Cancer immunotherapy focusing on the role of interleukins: A comprehensive and updated study. Pathol. Res. Pract 2023, 249, 154732. 37567033 10.1016/j.prp.2023.154732

[4] Brocker C; Thompson D; Matsumoto A; Nebert DW; Vasiliou V Evolutionary divergence and functions of the human interleukin (IL) gene family. Hum. Genomics 2010, 5, 30–55. 21106488 10.1186/1479-7364-5-1-30 PMC3390169

[5] Tsutsui H; Cai X; Hayashi S Interleukin-1 Family Cytokines in Liver Diseases. Mediat. Inflamm 2015, 2015, 630265. 10.1155/2015/630265 PMC4624893 26549942

[6] Schmitz J; Owyang A; Oldham E; Song Y; Murphy E; McClanahan TK; Zurawski G; Moshrefi M; Qin J; Li X; IL-33, an interleukin-1-like cytokine that signals via the IL-1 receptor-related protein ST2 and induces T helper type 2-associated cytokines. Immunity 2005, 23, 479–490. 16286016 10.1016/j.immuni.2005.09.015

[7] Baekkevold ES; Roussigné M; Yamanaka T; Johansen FE; Jahnsen FL; Amalric F; Brandtzaeg P; Erard M; Haraldsen G;Girard JP Molecular characterization of NF-HEV, a nuclear factor preferentially expressed in human high endothelial venules. Am. J. Pathol 2003, 163, 69–79. 12819012 10.1016/S0002-9440(10)63631-0 PMC1868188

[8] Gaurav R; Poole JA Interleukin (IL)-33 immunobiology in asthma and airway inflammatory diseases. J. Asthma 2022, 59, 2530–2538. 34928757 10.1080/02770903.2021.2020815 PMC9234100

[9] Di Carmine S; Scott MM; McLean MH; McSorley HJ The role of interleukin-33 in organ fibrosis. Discov. Immunol 2022, 1, kyac006. 38566909 10.1093/discim/kyac006 PMC10917208

[10] Chen WY; Li LC; Wu YH; Yang JL; Tzeng HT Emerging Roles of Interleukin-33-responsive Kidney Group 2 Innate Lymphoid Cells in Acute Kidney Injury. Int. J. Mol. Sci 2020, 21, 1544. 32102434 10.3390/ijms21041544 PMC7073188

[11] Brunetti G; Barile B; Nicchia GP; Onorati F; Luciani GB; Galeone A The ST2/IL-33 Pathway in Adult and Paediatric Heart Disease and Transplantation. Biomedicines 2023, 11, 1676. 37371771 10.3390/biomedicines11061676 PMC10296498

[12] De la Fuente M; MacDonald TT; Hermoso MA The IL-33/ST2 axis: Role in health and disease. Cytokine Growth Factor Rev. 2015, 26, 615–623. 26271893 10.1016/j.cytogfr.2015.07.017

[13] Yi XM; Lian H; Li S Signaling and functions of interleukin-33 in immune regulation and diseases. Cell Insight 2022, 1, 100042. 37192860 10.1016/j.cellin.2022.100042 PMC10120307

[14] Jia Z; Guo M; Ge X; Chen F; Lei P IL-33/ST2 Axis: A Potential Therapeutic Target in Neurodegenerative Diseases. Biomolecules 2023, 13, 1494. 37892176 10.3390/biom13101494 PMC10605306

[15] Sun J; Xia Y; Zhang D; Yu Z; Ning Y; Tan Z Knowledge mapping of interleukin-33: A bibliometric study. Am. J. Transl. Res 2023, 15, 914–931. 36915735 PMC10006773

[16] Okunogbe A; Nugent R; Spencer G; Powis J; Ralston J; Wilding J Economic impacts of overweight and obesity: Current and future estimates for 161 countries. BMJ Glob. Health 2022, 7, e009773. 10.1136/bmjgh-2022-009773 PMC9494015 36130777

[17] World Health Organization. World Health Statitiscs 2024: Monitoring Health for the SDGs, Sustainable Development Goals; WHO: Geneva, Switzerland, 2024.

[18] United States Cancer Statistics. 2024. Available online: https://www.cdc.gov/cancer/dataviz (accessed on 28 August 2024).

[19] Arnold M; Pandeya N; Byrnes G; Renehan PAG; Stevens GA; Ezzati PM; Ferlay J; Miranda JJ; Romieu I; Dikshit R; Global burden of cancer attributable to high body-mass index in 2012: A population-based study. Lancet Oncol. 2015, 16, 36–46. 25467404 10.1016/S1470-2045(14)71123-4 PMC4314462

[20] Hasan A; Kochumon S; Al-Ozairi E; Tuomilehto J; Ahmad R Association between Adipose Tissue Interleukin-33 and Immunometabolic Markers in Individuals with Varying Degrees of Glycemia. Dis. Markers 2019, 2019, 7901062. 31073344 10.1155/2019/7901062 PMC6470453

[21] Ferrante AW The immune cells in adipose tissue. Diabetes Obes. Metab 2013, 15 (Suppl. S3), S34–S38. 10.1111/dom.12154 PMC3777665 24003919

[22] de Oliveira MFA; Talvani A; Rocha-Vieira E IL-33 in obesity: Where do we go from here? Inflamm. Res 2019, 68, 185–194. 30656387 10.1007/s00011-019-01214-2

[23] Xiao P; Wan X; Cui B; Liu Y; Qiu C; Rong J; Zheng M; Song Y; Chen L; He J; Interleukin 33 in tumor microenvironment is crucial for the accumulation and function of myeloid-derived suppressor cells. Oncoimmunology 2016, 5, e1063772. 26942079 10.1080/2162402X.2015.1063772 PMC4760338

[24] Allegra A; Innao V; Tartarisco G; Pioggia G; Casciaro M; Musolino C; Gangemi S The ST2/Interleukin-33 Axis in Hematologic Malignancies: The IL-33 Paradox. Int. J. Mol. Sci 2019, 20, 5226. 31652497 10.3390/ijms20205226 PMC6834139

[25] Song M; Yang J; Di M; Hong Y; Pan Q; Du Y; Xiang T; Liu J; Tang Y; Wang Q; Alarmin IL-33 orchestrates antitumoral T cell responses to enhance sensitivity to 5-fluorouracil in colorectal cancer. Theranostics 2023, 13, 1649–1668. 37056569 10.7150/thno.80483 PMC10086207

[26] Chatrabnous N; Jafarzadeh A; Ghaderi A; Ariafar A; Aminizadeh N; Ghassabi F; Nemati M Association of elevated interleukin-33 serum levels with tumorstages in patients with prostate cancer. Eur. Cytokine Netw 2019, 30, 144–150. 32096476 10.1684/ecn.2019.0438

[27] Stojanovic B; Gajovic N; Jurisevic M; Stojanovic MD; Jovanovic M; Jovanovic I; Stojanovic BS; Milosevic B Decoding the IL-33/ST2 Axis: Its Impact on the Immune Landscape of Breast Cancer. Int. J. Mol. Sci 2023, 24, 14026. 37762328 10.3390/ijms241814026 PMC10531367

[28] Liu N; Chen J; Zhao Y; Zhang M; Piao L; Wang S; Yue Y Role of the IL-33/ST2 receptor axis in ovarian cancer progression. Oncol. Lett 2021, 22, 504. 33986865 10.3892/ol.2021.12765 PMC8114463

[29] Borovcanin MM; Vesic K Breast cancer in schizophrenia could be interleukin-33-mediated. World J. Psychiatry 2021, 11, 1065–1074. 34888174 10.5498/wjp.v11.i11.1065 PMC8613763

[30] Pati S; Irfan W; Jameel A; Ahmed S; Shahid RK Obesity and Cancer: A Current Overview of Epidemiology, Pathogenesis, Outcomes, and Management. Cancers 2023, 15, 485. 36672434 10.3390/cancers15020485 PMC9857053

[31] Garlanda C; Dinarello CA; Mantovani A The interleukin-1 family: Back to the future. Immunity 2013, 39, 1003–1018. 24332029 10.1016/j.immuni.2013.11.010 PMC3933951

[32] Carriere V; Roussel L; Ortega N; Lacorre DA; Americh L; Aguilar L; Bouche G; Girard JP IL-33, the IL-1-like cytokine ligand for ST2 receptor, is a chromatin-associated nuclear factor in vivo. Proc. Natl. Acad. Sci. USA 2007, 104, 282–287. 17185418 10.1073/pnas.0606854104 PMC1765450

[33] Cayrol C; Girard JP Interleukin-33 (IL-33): A nuclear cytokine from the IL-1 family. Immunol. Rev 2018, 281, 154–168.] 29247993 10.1111/imr.12619

[34] Liew FY; Girard JP; Turnquist HR Interleukin-33 in health and disease. Nat. Rev. Immunol 2016, 16, 676–689. 27640624 10.1038/nri.2016.95

[35] Luzina IG; Clerman A; Fishelevich R; Todd NW; Lockatell V; Atamas SP Identification of the IL-33 protein segment that controls subcellular localization, extracellular secretion, and functional maturation. Cytokine 2019, 119, 1–6. 30856600 10.1016/j.cyto.2019.02.015 PMC6535307

[36] Cayrol C; Girard JP The IL-1-like cytokine IL-33 is inactivated after maturation by caspase-1. Proc. Natl. Acad. Sci. USA 2009, 106, 9021–9026.. 19439663 10.1073/pnas.0812690106 PMC2690027

[37] Liu X; Hammel M; He Y; Tainer JA; Jeng US; Zhang L; Wang S; Wang X Structural insights into the interaction of IL-33 with its receptors. Proc. Natl. Acad. Sci. USA 2013, 110, 14918–14923. 23980170 10.1073/pnas.1308651110 PMC3773798

[38] Lingel A; Weiss TM; Niebuhr M; Pan B; Appleton BA; Wiesmann C; Bazan JF; Fairbrother WJ Structure of IL-33 and its interaction with the ST2 and IL-1RAcP receptors--insight into heterotrimeric IL-1 signaling complexes. Structure 2009, 17, 1398–1410. 19836339 10.1016/j.str.2009.08.009 PMC2766095

[39] Iwahana H; Yanagisawa K; Ito-Kosaka A; Kuroiwa K; Tago K; Komatsu N; Katashima R; Itakura M; Tominaga S Different promoter usage and multiple transcription initiation sites of the interleukin-1 receptor-related human ST2 gene in UT-7 and TM12 cells. Eur. J. Biochem 1999, 264, 397–406. 10491084 10.1046/j.1432-1327.1999.00615.x

[40] Klemenz R; Hoffmann S; Werenskiold AK Serum- and oncoprotein-mediated induction of a gene with sequence similarity to the gene encoding carcinoembryonic antigen. Proc. Natl. Acad. Sci. USA 1989, 86, 5708–5712. 2527364 10.1073/pnas.86.15.5708 PMC297699

[41] Tominaga S. A putative protein of a growth specific cDNA from BALB/c-3T3 cells is highly similar to the extracellular portion of mouse interleukin 1 receptor. FEBS Lett. 1989, 258, 301–304. 2532153 10.1016/0014-5793(89)81679-5

[42] Gächter T; Werenskiold AK; Klemenz R Transcription of the interleukin-1 receptor-related T1 gene is initiated at different promoters in mast cells and fibroblasts. J. Biol. Chem 1996, 271, 124–129. 8550546 10.1074/jbc.271.1.124

[43] Kakkar R; Lee RT The IL-33/ST2 pathway: Therapeutic target and novel biomarker. Nat. Rev. Drug Discov 2008, 7, 827–840. 18827826 10.1038/nrd2660 PMC4277436

[44] Mariotti FR; Supino D; Landolina N; Garlanda C; Mantovani A; Moretta L; Maggi E IL-1R8: A molecular brake of anti-tumor and anti-viral activity of NK cells and ILC. Semin. Immunol 2023, 66, 101712. 36753974 10.1016/j.smim.2023.101712

[45] Bulek K; Swaidani S; Qin J; Lu Y; Gulen MF; Herjan T; Min B; Kastelein RA; Aronica M; Kosz-Vnenchak M; The essential role of single Ig IL-1 receptor-related molecule/Toll IL-1R8 in regulation of Th2 immune response. J. Immunol 2009, 182, 2601–2609. 19234154 10.4049/jimmunol.0802729 PMC2891188

[46] Xu D; Chan WL; Leung BP; Huang F; Wheeler R; Piedrafita D; Robinson JH; Liew FY Selective expression of a stable cell surface molecule on type 2 but not type 1 helper T cells. J. Exp. Med 1998, 187, 787–794. 9480988 10.1084/jem.187.5.787 PMC2212173

[47] Ali S; Huber M; Kollewe C; Bischoff SC; Falk W; Martin MU IL-1 receptor accessory protein is essential for IL-33-induced activation of T lymphocytes and mast cells. Proc. Natl. Acad. Sci. USA 2007, 104, 18660–18665. 18003919 10.1073/pnas.0705939104 PMC2141833

[48] Chackerian AA; Oldham ER; Murphy EE; Schmitz J; Pflanz S; Kastelein RA IL-1 receptor accessory protein and ST2 comprise the IL-33 receptor complex. J. Immunol 2007, 179, 2551–2555. 17675517 10.4049/jimmunol.179.4.2551

[49] Cayrol C; Girard JP IL-33: An alarmin cytokine with crucial roles in innate immunity, inflammation and allergy. Curr. Opin. Immunol 2014, 31, 31–37. 25278425 10.1016/j.coi.2014.09.004

[50] He PY; Wu MY; Zheng LY; Duan Y; Fan Q; Zhu XM; Yao YM Interleukin-33/serum stimulation-2 pathway: Regulatory mechanisms and emerging implications in immune and inflammatory diseases. Cytokine Growth Factor Rev. 2024, 76, 112–126. 38155038 10.1016/j.cytogfr.2023.12.001

[51] Seki K; Sanada S; Kudinova AY; Steinhauser ML; Handa V; Gannon J; Lee RT Interleukin-33 prevents apoptosis and improves survival after experimental myocardial infarction through ST2 signaling. Circ. Heart Fail 2009, 2, 684–691. 19919994 10.1161/CIRCHEARTFAILURE.109.873240

[52] Zarezadeh Mehrabadi A; Shahba F; Khorramdelazad H; Aghamohammadi N; Karimi M; Bagherzadeh K; Khoshmirsafa M; Massoumi R; Falak R Interleukin-1 receptor accessory protein (IL-1RAP): A magic bullet candidate for immunotherapy of human malignancies. Crit. Rev. Oncol. Hematol 2024, 193, 104200. 37981104 10.1016/j.critrevonc.2023.104200

[53] Choi YS; Choi HJ; Min JK; Pyun BJ; Maeng YS; Park H; Kim J; Kim YM; Kwon YG Interleukin-33 induces angiogenesis and vascular permeability through ST2/TRAF6-mediated endothelial nitric oxide production. Blood 2009, 114, 3117–3126. 19661270 10.1182/blood-2009-02-203372

[54] Artru F; Bou Saleh M; Maggiotto F; Lassailly G; Ningarhari M; Demaret J; Ntandja-Wandji LC; Pais de Barros JP; Labreuche J; Drumez E; IL-33/ST2 pathway regulates neutrophil migration and predicts outcome in patients with severe alcoholic hepatitis. J. Hepatol 2020, 72, 1052–1061 31953139 10.1016/j.jhep.2019.12.017

[55] Zeyda M; Wernly B; Demyanets S; Kaun C; Hämmerle M; Hantusch B; Schranz M; Neuhofer A; Itariu BK; Keck M; Severe obesity increases adipose tissue expression of interleukin-33 and its receptor ST2, both predominantly detectable in endothelial cells of human adipose tissue. Int. J. Obes 2013, 37, 658–665. 10.1038/ijo.2012.118 22828942

[56] Kwon JW; Seok SH; Kim S; An HW; Choudhury AD; Woo SH; Oh JS; Kim JK; Voon DC; Kim DY; A synergistic partnership between IL-33/ST2 and Wnt pathway through Bcl-xL drives gastric cancer stemness and metastasis. Oncogene 2023, 42, 501–515. 36526851 10.1038/s41388-022-02575-5

[57] Cayrol C; Girard JP Interleukin-33 (IL-33): A critical review of its biology and the mechanisms involved in its release as a potent extracellular cytokine. Cytokine 2022, 156, 155891. 35640416 10.1016/j.cyto.2022.155891

[58] Göpfert C; Andreas N; Weber F; Häfner N; Yakovleva T; Gaestel M; Kamradt T; Drube S The p38-MK2/3 Module Is Critical for IL-33-Induced Signaling and Cytokine Production in Dendritic Cells. J. Immunol 2018, 200, 1198–1206. 29288203 10.4049/jimmunol.1700727

[59] Ishiguro N; Moriyama M; Furusho K; Furukawa S; Shibata T; Murakami Y; Chinju A; Haque ASMR; Gion Y; Ohta M; Activated M2 Macrophages Contribute to the Pathogenesis of IgG4-Related Disease via Toll-like Receptor 7/Interleukin-33 Signaling. Arthritis Rheumatol. 2020, 72, 166–178. 31339007 10.1002/art.41052 PMC6972995

[60] Löhning M; Stroehmann A; Coyle AJ; Grogan JL; Lin S; Gutierrez-Ramos JC; Levinson D; Radbruch A; Kamradt T T1/ST2 is preferentially expressed on murine Th2 cells, independent of interleukin 4, interleukin 5, and interleukin 10, and important for Th2 effector function. Proc. Natl. Acad. Sci. USA 1998, 95, 6930–6935. 9618516 10.1073/pnas.95.12.6930 PMC22690

[61] Weinberg EO; Shimpo M; De Keulenaer GW; MacGillivray C; Tominaga S; Solomon SD; Rouleau JL; Lee RT Expression and regulation of ST2, an interleukin-1 receptor family member, in cardiomyocytes and myocardial infarction. Circulation 2002, 106, 2961–2966. 12460879 10.1161/01.CIR.0000038705.69871.D9 PMC1460012

[62] Griesenauer B; Paczesny S The ST2/IL-33 Axis in Immune Cells during Inflammatory Diseases. Front. Immunol 2017, 8, 475. 28484466 10.3389/fimmu.2017.00475 PMC5402045

[63] Neill DR; Wong SH; Bellosi A; Flynn RJ; Daly M; Langford TK; Bucks C; Kane CM; Fallon PG; Pannell R; Nuocytes represent a new innate effector leukocyte that mediates type-2 immunity. Nature 2010, 464, 1367–1370. 20200518 10.1038/nature08900 PMC2862165

[64] Schiering C; Krausgruber T; Chomka A; Fröhlich A; Adelmann K; Wohlfert EA; Pott J; Griseri T; Bollrath J; Hegazy AN; The alarmin IL-33 promotes regulatory T-cell function in the intestine. Nature 2014, 513, 564–568. 25043027 10.1038/nature13577 PMC4339042

[65] Pastille E; Wasmer MH; Adamczyk A; Vu VP; Mager LF; Phuong NNT; Palmieri V; Simillion C; Hansen W; Kasper S; The IL-33/ST2 pathway shapes the regulatory T cell phenotype to promote intestinal cancer. Mucosal Immunol. 2019, 12, 990–1003. 31165767 10.1038/s41385-019-0176-y PMC7746527

[66] Bourgeois E; Van LP; Samson M; Diem S; Barra A; Roga S; Gombert M; Schneider E; Dy M; Gourdy P; The pro-Th2 cytokine IL-33 directly interacts with invariant NKT and NK cells to induce IFN-γ production. Eur. J. Immunol 2009, 39, 1046–1055. 19266498 10.1002/eji.200838575

[67] Zou L; Dang W; Tao Y; Zhao H; Yang B; Xu X; Li Y THE IL-33/ST2 Axis Promotes Acute Respiratory Distress Syndrome by Natural Killer T Cells. Shock 2023, 59, 902–911. 36870074 10.1097/SHK.0000000000002114 PMC10227934

[68] Baumann C; Bonilla WV; Fröhlich A; Helmstetter C; Peine M; Hegazy AN; Pinschewer DD; Löhning M T-bet- and STAT4-dependent IL-33 receptor expression directly promotes antiviral Th1 cell responses. Proc. Natl. Acad. Sci. USA 2015, 112, 4056–4061. 25829541 10.1073/pnas.1418549112 PMC4386370

[69] Dwyer GK; D’Cruz LM; Turnquist HR Emerging Functions of IL-33 in Homeostasis and Immunity. Annu. Rev. Immunol 2022, 40, 15–43. 34985928 10.1146/annurev-immunol-101320-124243

[70] Brunner TM; Serve S; Marx AF; Fadejeva J; Saikali P; Dzamukova M; Durán-Hernández N; Kommer C; Heinrich F; Durek P; A type 1 immunity-restricted promoter of the IL-33 receptor gene directs antiviral T-cell responses. Nat. Immunol 2024, 25, 256–267. 38172258 10.1038/s41590-023-01697-6 PMC10834369

[71] Milovanovic M; Volarevic V; Radosavljevic G; Jovanovic I; Pejnovic N; Arsenijevic N; Lukic ML IL-33/ST2 axis in inflammation and immunopathology. Immunol. Res 2012, 52, 89–99. [22392053 10.1007/s12026-012-8283-9

[72] O’Donnell C; Mahmoud A; Keane J; Murphy C; White D; Carey S; O’Riordain M; Bennett MW; Brint E; Houston A An antitumorigenic role for the IL-33 receptor, ST2L, in colon cancer. Br. J. Cancer 2016, 114, 37–43. 26679377 10.1038/bjc.2015.433 PMC4716545

[73] Kurowska-Stolarska M; Kewin P; Murphy G; Russo RC; Stolarski B; Garcia CC; Komai-Koma M; Pitman N; Li Y; Niedbala W; IL-33 induces antigen-specific IL-5+ T cells and promotes allergic-induced airway inflammation independent of IL-4. J. Immunol 2008, 181, 4780–4790. 18802081 10.4049/jimmunol.181.7.4780

[74] Kokubo K; Onodera A; Kiuchi M; Tsuji K; Hirahara K; Nakayama T Conventional and pathogenic Th2 cells in inflammation, tissue repair, and fibrosis. Front. Immunol 2022, 13, 945063. 36016937 10.3389/fimmu.2022.945063 PMC9395650

[75] Stark JM; Tibbitt CA; Coquet JM The Metabolic Requirements of Th2 Cell Differentiation. Front. Immunol 2019, 10, 02318. 10.3389/fimmu.2019.02318 PMC6776632 31611881

[76] Setrerrahmane S; Xu H Tumor-related interleukins: Old validated targets for new anti-cancer drug development. Mol. Cancer 2017, 16, 153. 28927416 10.1186/s12943-017-0721-9 PMC5606116

[77] Joshi BH; Leland P; Lababidi S; Varrichio F; Puri RK Interleukin-4 receptor alpha overexpression in human bladder cancer correlates with the pathological grade and stage of the disease. Cancer Med. 2014, 3, 1615–1628.] 25208941 10.1002/cam4.330 PMC4298388

[78] Prokopchuk O; Liu Y; Henne-Bruns D; Kornmann M Interleukin-4 enhances proliferation of human pancreatic cancer cells: Evidence for autocrine and paracrine actions. Br. J. Cancer 2005, 92, 921–928. 15714203 10.1038/sj.bjc.6602416 PMC2361902

[79] Todaro M; Lombardo Y; Francipane MG; Alea MP; Cammareri P; Iovino F; Di Stefano AB; Di Bernardo C; Agrusa A; Condorelli G; Apoptosis resistance in epithelial tumors is mediated by tumor-cell-derived interleukin-4. Cell Death Differ. 2008, 15, 762–772. 18202702 10.1038/sj.cdd.4402305

[80] Brightling CE; Desai D; Pavord ID Cytokine-Specific Therapy in Asthma. In Middleton’s Allergy, 8th ed.; Elsevier: Amsterdam, The Netherlands, 2014; pp. 1491–1502.

[81] Pelaia C; Paoletti G; Puggioni F; Racca F; Pelaia G; Canonica GW; Heffler E Interleukin-5 in the Pathophysiology of Severe Asthma. Front. Physiol 2019, 10, 1514. 31920718 10.3389/fphys.2019.01514 PMC6927944

[82] Gour N; Wills-Karp M IL-4 and IL-13 signaling in allergic airway disease. Cytokine 2015, 75, 68–78. 26070934 10.1016/j.cyto.2015.05.014 PMC4532591

[83] Seibold MA Interleukin-13 Stimulation Reveals the Cellular and Functional Plasticity of the Airway Epithelium. Ann. Am. Thorac. Soc 2018, 15 (Suppl. S2), S98–S102. 29676620 10.1513/AnnalsATS.201711-868MG PMC5955044

[84] Doran E; Cai F; Holweg CTJ; Wong K; Brumm J; Arron JR Interleukin-13 in Asthma and Other Eosinophilic Disorders. Front. Med 2017, 4, 139. 10.3389/fmed.2017.00139 PMC5627038 29034234

[85] Knudson KM; Hwang S; McCann MS; Joshi BH; Husain SR; Puri RK Recent Advances in IL-13Rα2-Directed Cancer Immunotherapy. Front. Immunol 2022, 13, 878365. 35464460 10.3389/fimmu.2022.878365 PMC9023787

[86] Kirichenko TV; Markina YV; Bogatyreva AI; Tolstik TV; Varaeva YR; Starodubova AV The Role of Adipokines in Inflammatory Mechanisms of Obesity. Int. J. Mol. Sci 2022, 23, 14982. 36499312 10.3390/ijms232314982 PMC9740598

[87] Schmidt FM; Weschenfelder J; Sander C; Minkwitz J; Thormann J; Chittka T; Mergl R; Kirkby KC; Faßhauer M; Stumvoll M; Inflammatory Cytokines in General and Central Obesity and Modulating Effects of Physical Activity. PLoS ONE 2015, 10, e0121971. 25781614 10.1371/journal.pone.0121971 PMC4363366

[88] Kim JW; Kim JH; Lee YJ The Role of Adipokines in Tumor Progression and Its Association with Obesity. Biomedicines 2024, 12, 97. 38255203 10.3390/biomedicines12010097 PMC10813163

[89] Gesta S; Tseng YH; Kahn CR Developmental origin of fat: Tracking obesity to its source. Cell 2007, 131, 242–256. 17956727 10.1016/j.cell.2007.10.004

[90] Frontini A; Cinti S Distribution and development of brown adipocytes in the murine and human adipose organ. Cell Metab. 2010, 11, 253–256. 20374956 10.1016/j.cmet.2010.03.004

[91] Rosen ED; Spiegelman BM What we talk about when we talk about fat. Cell 2014, 156, 20–44. 24439368 10.1016/j.cell.2013.12.012 PMC3934003

[92] Lee MJ; Wu Y; Fried SK Adipose tissue heterogeneity: Implication of depot differences in adipose tissue for obesity complications. Mol. Asp. Med 2013, 34, 1–11. 10.1016/j.mam.2012.10.001 PMC3549425 23068073

[93] Michailidou Z; Gomez-Salazar M; Alexaki VI Innate Immune Cells in the Adipose Tissue in Health and Metabolic Disease. J. Innate Immun 2022, 14, 4–30. 33849008 10.1159/000515117 PMC8787575

[94] Man K; Kallies A; Vasanthakumar A Resident and migratory adipose immune cells control systemic metabolism and thermogenesis. Cell Mol. Immunol 2022, 19, 421–431. 34837070 10.1038/s41423-021-00804-7 PMC8891307

[95] Mathis D. Immunological goings-on in visceral adipose tissue. Cell Metab. 2013, 17, 851–859. 23747244 10.1016/j.cmet.2013.05.008 PMC4264591

[96] Deiuliis J; Shah Z; Shah N; Needleman B; Mikami D; Narula V; Perry K; Hazey J; Kampfrath T; Kollengode M; Visceral adipose inflammation in obesity is associated with critical alterations in tregulatory cell numbers. PLoS ONE 2011, 6, e16376. 21298111 10.1371/journal.pone.0016376 PMC3027666

[97] Molofsky AB; Van Gool F; Liang HE; Van Dyken SJ; Nussbaum JC; Lee J; Bluestone JA; Locksley RM Interleukin-33 and Interferon-γ Counter-Regulate Group 2 Innate Lymphoid Cell Activation during Immune Perturbation. Immunity 2015, 43, 161–174. 26092469 10.1016/j.immuni.2015.05.019 PMC4512852

[98] Odegaard JI; Lee MW; Sogawa Y; Bertholet AM; Locksley RM; Weinberg DE; Kirichok Y; Deo RC; Chawla A Perinatal Licensing of Thermogenesis by IL-33 and ST2. Cell 2016, 166, 841–854. 27453471 10.1016/j.cell.2016.06.040 PMC4985267

[99] Li Q; Li D; Zhang X; Wan Q; Zhang W; Zheng M; Zou L; Elly C; Lee JH; Liu YC E3 Ligase VHL Promotes Group 2 Innate Lymphoid Cell Maturation and Function via Glycolysis Inhibition and Induction of Interleukin-33 Receptor. Immunity 2018, 48, 258–270.e5. 29452935 10.1016/j.immuni.2017.12.013 PMC5828523

[100] Miller AM; Asquith DL; Hueber AJ; Anderson LA; Holmes WM; McKenzie AN; Xu D; Sattar N; McInnes IB; Liew FY Interleukin-33 induces protective effects in adipose tissue inflammation during obesity in mice. Circ. Res 2010, 107, 650–658. 20634488 10.1161/CIRCRESAHA.110.218867 PMC4254700

[101] Mahlakõiv T; Flamar AL; Johnston LK; Moriyama S; Putzel GG; Bryce PJ; Artis D Stromal cells maintain immune cell homeostasis in adipose tissue via production of interleukin-33. Sci. Immunol 2019, 4, eaax0416. 31053655 10.1126/sciimmunol.aax0416 PMC6766755

[102] Brestoff JR; Kim BS; Saenz SA; Stine RR; Monticelli LA; Sonnenberg GF; Thome JJ; Farber DL; Lutfy K; Seale P; Group 2 innate lymphoid cells promote beiging of white adipose tissue and limit obesity. Nature 2015, 519, 242–246. 25533952 10.1038/nature14115 PMC4447235

[103] Lee MW; Odegaard JI; Mukundan L; Qiu Y; Molofsky AB; Nussbaum JC; Yun K; Locksley RM; Chawla A Activated type 2 innate lymphoid cells regulate beige fat biogenesis. Cell 2015, 160, 74–87. 25543153 10.1016/j.cell.2014.12.011 PMC4297518

[104] Wang L; Luo Y; Luo L; Wu D; Ding X; Zheng H; Wu H; Liu B; Yang X; Silva F; Adiponectin restrains ILC2 activation by AMPK-mediated feedback inhibition of IL-33 signaling. J. Exp. Med 2021, 218, e20191054. 33104171 10.1084/jem.20191054 PMC7590510

[105] Zhang S; Gang X; Yang S; Cui M; Sun L; Li Z; Wang G The Alterations in and the Role of the Th17/Treg Balance in Metabolic Diseases. Front. Immunol 2021, 12, 678355. 34322117 10.3389/fimmu.2021.678355 PMC8311559

[106] Wang Q; Wang Y; Xu D The roles of T cells in obese adipose tissue inflammation. Adipocyte 2021, 10, 435–445. 34515616 10.1080/21623945.2021.1965314 PMC8463033

[107] Schmidt V; Hogan AE; Fallon PG; Schwartz C Obesity-Mediated Immune Modulation: One Step Forward, (Th)2 Steps Back. Front. Immunol 2022, 13, 932893. 35844529 10.3389/fimmu.2022.932893 PMC9279727

[108] Zhao XY; Zhou L; Chen Z; Ji Y; Peng X; Qi L; Li S; Lin JD The obesity-induced adipokine sST2 exacerbates adipose T. Sci. Adv 2020, 6, eaay6191. 32426492 10.1126/sciadv.aay6191 PMC7220368

[109] Yang Q; Li G; Zhu Y; Liu L; Chen E; Turnquist H; Zhang X; Finn OJ; Chen X; Lu B IL-33 synergizes with TCR and IL-12 signaling to promote the effector function of CD8+ T cells. Eur. J. Immunol 2011, 41, 3351–3360. 21887788 10.1002/eji.201141629 PMC3332117

[110] Smithgall MD; Comeau MR; Yoon BR; Kaufman D; Armitage R; Smith DE IL-33 amplifies both Th1- and Th2-type responses through its activity on human basophils, allergen-reactive Th2 cells, iNKT and NK cells. Int. Immunol 2008, 20, 1019–1030. 18550585 10.1093/intimm/dxn060

[111] Arrizabalaga L; Risson A; Ezcurra-Hualde M; Aranda F; Berraondo P Unveiling the multifaceted antitumor effects of interleukin 33. Front. Immunol 2024, 15, 1425282. 38881897 10.3389/fimmu.2024.1425282 PMC11176530

[112] Anderson NM; Simon MC The tumor microenvironment. Curr. Biol 2020, 30, R921–R925. 32810447 10.1016/j.cub.2020.06.081 PMC8194051

[113] Truffi M; Sorrentino L; Corsi F Fibroblasts in the Tumor Microenvironment. Adv. Exp. Med. Biol 2020, 1234, 15–29. 32040852 10.1007/978-3-030-37184-5_2

[114] Lu B; Yang M; Wang Q Interleukin-33 in tumorigenesis, tumor immune evasion, and cancer immunotherapy. J. Mol. Med 2016, 94, 535–543. 26922618 10.1007/s00109-016-1397-0

[115] Peng L; Sun W; Wei F; Chen L; Wen W Interleukin-33 modulates immune responses in cutaneous melanoma in a contextspecific way. Aging 2021, 13, 6740–6751. 33621202 10.18632/aging.202531 PMC7993738

[116] Schuijs MJ; Png S; Richard AC; Tsyben A; Hamm G; Stockis J; Garcia C; Pinaud S; Nicholls A; Ros XR; ILC2-driven innate immune checkpoint mechanism antagonizes NK cell antimetastatic function in the lung. Nat. Immunol 2020, 21, 998–1009. 32747815 10.1038/s41590-020-0745-y PMC7116357

[117] Gao K; Li X; Zhang L; Bai L; Dong W; Shi G; Xia X; Wu L Transgenic expression of IL-33 activates CD8(+) T cells and NK cells and inhibits tumor growth and metastasis in mice. Cancer Lett. 2013, 335, 463–471. 23499895 10.1016/j.canlet.2013.03.002

[118] Gao X; Wang X; Yang Q; Zhao X; Wen W; Li G; Lu J; Qin W; Qi Y; Xie F; Tumoral expression of IL-33 inhibits tumor growth and modifies the tumor microenvironment through CD8+ T and NK cells. J. Immunol 2015, 194, 438–445. 25429071 10.4049/jimmunol.1401344 PMC4272901

[119] Lucarini V; Ziccheddu G; Macchia I; La Sorsa V; Peschiaroli F; Buccione C; Sistigu A; Sanchez M; Andreone S; D’Urso MT; IL-33 restricts tumor growth and inhibits pulmonary metastasis in melanoma-bearing mice through eosinophils. Oncoimmunology 2017, 6, e1317420. 28680750 10.1080/2162402X.2017.1317420 PMC5486175

[120] Dominguez D; Ye C; Geng Z; Chen S; Fan J; Qin L; Long A; Wang L; Zhang Z; Zhang Y; Exogenous IL-33 Restores Dendritic Cell Activation and Maturation in Established Cancer. J. Immunol 2017, 198, 1365–1375. 28011934 10.4049/jimmunol.1501399 PMC5263113

[121] Briukhovetska D; Dörr J; Endres S; Libby P; Dinarello CA; Kobold S Interleukins in cancer: From biology to therapy. Nat. Rev. Cancer 2021, 21, 481–499. 34083781 10.1038/s41568-021-00363-z PMC8173513

[122] Hatzioannou A; Banos A; Sakelaropoulos T; Fedonidis C; Vidali MS; Köhne M; Händler K; Boon L; Henriques A; Koliaraki V; An intrinsic role of IL-33 in T. Nat. Immunol 2020, 21, 75–85. 31844326 10.1038/s41590-019-0555-2 PMC7030950

[123] Yeoh WJ; Vu VP; Krebs P IL-33 biology in cancer: An update and future perspectives. Cytokine 2022, 157, 155961. 35843125 10.1016/j.cyto.2022.155961

[124] Alvarez F; Fritz JH; Piccirillo CA Pleiotropic Effects of IL-33 on CD4+ T Cell Differentiation and Effector Functions. Rontiers Immunol. 2019, 10, 522. 10.3389/fimmu.2019.00522 PMC6435597 30949175

[125] Zhou Z; Yan F; Liu O Interleukin (IL)-33: An orchestrator of immunity from host defence to tissue homeostasis. Clin. Transl. Immunol 2020, 9, e1146. 10.1002/cti2.1146 PMC7299676 32566227

[126] Andreone S; Spadaro F; Buccione C; Mancini J; Tinari A; Sestili P; Gambardella AR; Lucarini V; Ziccheddu G; Parolini I; IL-33 Promotes CD11b/CD18-Mediated Adhesion of Eosinophils to Cancer Cells and Synapse-Polarized Degranulation Leading to Tumor Cell Killing. Cancers 2019, 11, 1664. 31717819 10.3390/cancers11111664 PMC6895824

[127] Marichal T; Tsai M; Galli SJ Mast cells: Potential positive and negative roles in tumor biology. Cancer Immunol. Res 2013, 1, 269–279. 24777963 10.1158/2326-6066.CIR-13-0119

[128] Ribatti D. Mast Cells and Resistance to Immunotherapy in Cancer. Arch. Immunol. Ther. Exp 2023, 71, 11. 10.1007/s00005-023-00676-x PMC10085948 37038035

[129] Lv Y; Tian W; Teng Y; Wang P; Zhao Y; Li Z; Tang S; Chen W; Xie R; Lü M; Tumor-infiltrating mast cells stimulate ICOS. J. Adv. Res 2024, 57, 149–162. 37086778 10.1016/j.jare.2023.04.013 PMC10918354

[130] Andreone S; Gambardella AR; Mancini J; Loffredo S; Marcella S; La Sorsa V; Varricchi G; Schiavoni G; Mattei F Anti-Tumorigenic Activities of IL-33: A Mechanistic Insight. Front. Immunol 2020, 11, 571593. 33329534 10.3389/fimmu.2020.571593 PMC7734277

[131] Schneider E; Petit-Bertron AF; Bricard R; Levasseur M; Ramadan A; Girard JP; Herbelin A; Dy M IL-33 activates unprimed murine basophils directly in vitro and induces their in vivo expansion indirectly by promoting hematopoietic growth factor production. J. Immunol 2009, 183, 3591–3597. 19684081 10.4049/jimmunol.0900328

[132] Pecaric-Petkovic T; Didichenko SA; Kaempfer S; Spiegl N; Dahinden CA Human basophils and eosinophils are the direct target leukocytes of the novel IL-1 family member IL-33. Blood 2009, 113, 1526–1534. 18955562 10.1182/blood-2008-05-157818 PMC2644080

[133] Rivellese F; Suurmond J; de Paulis A; Marone G; Huizinga TW; Toes RE IgE and IL-33-mediated triggering of human basophils inhibits TLR4-induced monocyte activation. Eur. J. Immunol 2014, 44, 3045–3055. 25070188 10.1002/eji.201444731

[134] Afferni C; Buccione C; Andreone S; Galdiero MR; Varricchi G; Marone G; Mattei F; Schiavoni G The Pleiotropic Immunomodulatory Functions of IL-33 and Its Implications in Tumor Immunity. Front. Immunol 2018, 9, 2601. 30483263 10.3389/fimmu.2018.02601 PMC6242976

[135] Prete AD; Salvi V; Soriani A; Laffranchi M; Sozio F; Bosisio D; Sozzani S Dendritic cell subsets in cancer immunity and tumor antigen sensing. Cell. Mol. Immunol 2023, 20, 432–447. 36949244 10.1038/s41423-023-00990-6 PMC10203372

[136] Xu H; Li D; Ma J; Zhao Y; Xu L; Tian R; Liu Y; Sun L; Su J The IL-33/ST2 axis affects tumor growth by regulating mitophagy in macrophages and reprogramming their polarization. Cancer Biol. Med 2021, 18, 172–183. 33628592 10.20892/j.issn.2095-3941.2020.0211 PMC7877183

[137] Chen J; Zhao Y; Jiang Y; Gao S; Wang Y; Wang D; Wang A; Yi H; Gu R; Yi Q; Interleukin-33 Contributes to the Induction of Th9 Cells and Antitumor Efficacy by Dectin-1-Activated Dendritic Cells. Front. Immunol 2018, 9, 1787. 30108595 10.3389/fimmu.2018.01787 PMC6079242

[138] Zhang X; Chen W; Zeng P; Xu J; Diao H The Contradictory Role of Interleukin-33 in Immune Cells and Tumor Immunity. Cancer Manag. Res 2020, 12, 7527–7537. 32904627 10.2147/CMAR.S262745 PMC7457384

[139] Liu X; Li L; Si F; Huang L; Zhao Y; Zhang C; Hoft DF; Peng G NK and NKT cells have distinct properties and functions in cancer. Oncogene 2021, 40, 4521–4537. 34120141 10.1038/s41388-021-01880-9 PMC12416827

[140] Choi MR; Sosman JA; Zhang B The Janus Face of IL-33 Signaling in Tumor Development and Immune Escape. Cancers 2021, 13, 3281. 34209038 10.3390/cancers13133281 PMC8268428

[141] Komai-Koma M; Wang E; Kurowska-Stolarska M; Li D; McSharry C; Xu D Interleukin-33 promoting Th1 lymphocyte differentiation dependents on IL-12. Immunobiology 2016, 221, 412–417. 26688508 10.1016/j.imbio.2015.11.013 PMC4731778

[142] Wu X; Tian J; Wang S Insight Into Non-Pathogenic Th17 Cells in Autoimmune Diseases. Front. Immunol 2018, 9, 1112. 29892286 10.3389/fimmu.2018.01112 PMC5985293

[143] Xing J; Man C; Liu Y; Zhang Z; Peng H Factors impacting the benefits and pathogenicity of Th17 cells in the tumor microenvironment. Front. Immunol 2023, 14, 1224269. 37680632 10.3389/fimmu.2023.1224269 PMC10481871

[144] Pascual-Reguant A; Bayat Sarmadi J; Baumann C; Noster R; Cirera-Salinas D; Curato C; Pelczar P; Huber S; Zielinski CE; Löhning M; T_H17_ cells express ST_2_ and are controlled by the alarmin IL-_33_ in the small intestine. Mucosal Immunol. 2017, 10, 1431–1442. 28198366 10.1038/mi.2017.5

[145] Guo H; Bossila EA; Ma X; Zhao C; Zhao Y Dual Immune Regulatory Roles of Interleukin-33 in Pathological Conditions. Cells 2022, 11, 3237. 36291105 10.3390/cells11203237 PMC9600220

[146] Lei S; Jin J; Zhao X; Zhou L; Qi G; Yang J The role of IL-33/ST2 signaling in the tumor microenvironment and Treg immunotherapy. Exp. Biol. Med 2022, 247, 1810–1818. 10.1177/15353702221102094 PMC9679353 35733343

[147] Chen CC; Kobayashi T; Iijima K; Hsu FC; Kita H IL-33 dysregulates regulatory T cells and impairs established immunologic tolerance in the lungs. J. Allergy Clin. Immunol 2017, 140, 1351–1363.e1357. 28196763 10.1016/j.jaci.2017.01.015 PMC5554091

[148] Shani O; Vorobyov T; Monteran L; Lavie D; Cohen N; Raz Y; Tsarfaty G; Avivi C; Barshack I; Erez N Fibroblast-Derived IL33 Facilitates Breast Cancer Metastasis by Modifying the Immune Microenvironment and Driving Type 2 Immunity. Cancer Res. 2020, 80, 5317–5329. 33023944 10.1158/0008-5472.CAN-20-2116 PMC7611300

[149] Monteran L; Erez N The Dark Side of Fibroblasts: Cancer-Associated Fibroblasts as Mediators of Immunosuppression in the Tumor Microenvironment. Front. Immunol 2019, 10, 1835. 31428105 10.3389/fimmu.2019.01835 PMC6688105

[150] Chen SF; Nieh S; Jao SW; Wu MZ; Liu CL; Chang YC; Lin YS The paracrine effect of cancer-associated fibroblast-induced interleukin-33 regulates the invasiveness of head and neck squamous cell carcinoma. J. Pathol 2013, 231, 180–189. 23775566 10.1002/path.4226

[151] Peng L; Sun W; Chen L; Wen WP The Role of Interleukin-33 in Head and Neck Squamous Cell Carcinoma Is Determined by Its Cellular Sources in the Tumor Microenvironment. Front. Oncol 2020, 10, 588454. 33634017 10.3389/fonc.2020.588454 PMC7902021

[152] Andersson P; Yang Y; Hosaka K; Zhang Y; Fischer C; Braun H; Liu S; Yu G; Beyaert R; Chang M; Molecular mechanisms of IL-33-mediated stromal interactions in cancer metastasis. JCI Insight 2018, 3, e122375. 30333314 10.1172/jci.insight.122375 PMC6237443

[153] He Z; Chen L; Souto FO; Canasto-Chibuque C; Bongers G; Deshpande M; Harpaz N; Ko HM; Kelley K; Furtado GC; Epithelial-derived IL-33 promotes intestinal tumorigenesis in Apc. Sci. Rep 2017, 7, 5520. 28710436 10.1038/s41598-017-05716-z PMC5511216

[154] Chen L; Sun R; Xu J; Zhai W; Zhang D; Yang M; Yue C; Chen Y; Li S; Turnquist H; Tumor-Derived IL33 Promotes Tissue-Resident CD8. Cancer Immunol. Res 2020, 8, 1381–1392. ] 32917659 10.1158/2326-6066.CIR-19-1024 PMC7642190

[155] Yang M; Feng Y; Yue C; Xu B; Chen L; Jiang J; Lu B; Zhu Y Lower expression level of IL-33 is associated with poor prognosis of pulmonary adenocarcinoma. PLoS ONE 2018, 13, e0193428. 29499051 10.1371/journal.pone.0193428 PMC5834175

[156] Liu J; Shen JX; Hu JL; Huang WH; Zhang GJ Significance of interleukin-33 and its related cytokines in patients with breast cancers. Front. Immunol 2014, 5, 141. 24778632 10.3389/fimmu.2014.00141 PMC3985005

[157] Kim JY; Kim G; Lim SC; Choi HS IL-33-Induced Transcriptional Activation of LPIN1 Accelerates Breast Tumorigenesis. Cancers 2021, 13, 2174. 33946554 10.3390/cancers13092174 PMC8124251

[158] Kolb R; Sutterwala FS; Zhang W Obesity and cancer: Inflammation bridges the two. Curr. Opin. Pharmacol 2016, 29, 77–89. 27429211 10.1016/j.coph.2016.07.005 PMC4992602

